# PlantRep: a database of plant repetitive elements

**DOI:** 10.1007/s00299-021-02817-y

**Published:** 2022-01-03

**Authors:** Xizhi Luo, Shiyu Chen, Yu Zhang

**Affiliations:** 1 Shenzhen Branch, Guangdong Laboratory for Lingnan Modern Agriculture, Genome Analysis Laboratory of the Ministry of Agriculture, Agricultural Genomics Institute at Shenzhen, Chinese Academy of Agricultural Sciences, Shenzhen, 518124 China; 2 School of Agriculture, Sun Yat-sen University, Shenzhen, 518107 China

**Keywords:** Database of repeat sequences, Evolution of plant genome, Transposable element

## Abstract

**We re-annotated repeats of 459 plant genomes and released a new database: PlantRep (** http://www.plantrep.cn/ **). PlantRep sheds lights of repeat evolution and provides fundamental data for deep exploration of genome.**

**Supplementary Information:**

The online version contains supplementary material available at 10.1007/s00299-021-02817-y.

## Introduction

With the rapid release of genome, exploring the comparative genomics of repeats enabled us to elucidate how repeat sequences originated and amplified in different plant lineages. However, large-scale evolution analysis in plants takes a considerable amount of time and computing resources; hence, the comparative genomics and evolution analysis of transposons have only been conducted in a few selected plants (Baidouri and Panaud 2013; Elliott and Gregory 2015; Schaper and Anisimova 2015) and the current reference repeat databases only contain a few model plants (Jurka et al. 2005; Bao et al. 2015; Hubley et al. 2016). Besides, the repeat annotations carried out independently using heterogeneous pipelines cannot be used directly for comparative studies. To cope with this issue, we utilized a uniformed pipeline to re-annotate repeats for 459 plant genomes and compared the repeat sequences among the plant groups, including the composition, family diversity, genomic distribution, and evolutionary rate. The results provide a resource for the analysis and study of the repeat sequences in different lineages of plants.

## Results and discussion

We re-annotated repeats from 459 released plant genomes and generated 45.72 Gb seed alignments and 601,731 consensus sequences of repeats from de novo repeat annotation of each plant genome. The repeat libraries are available in our database PlantRep. Combined with the reference-based annotation, 206.04 Gb of 396,041,410 repeats were identified and categorized. We released this consensus repeat annotations (PlantRep) for the plant community as an updated resource for the future data-mining studies. Repeats in the PlantRep database were categorized with repeat types adapted from the existing eukaryotic transposable element classes and the Dfam database (Hubley et al. 2016; Wicker et al. 2007) (Supplementary Table 1c). Retrotransposon includes long terminal repeat (LTR), dictyostelium intermediate repeat sequence (DIRS), penelope (PLE), long interspersed nuclear element (LINE), and short LINE-dependent retroposons (SINE). DNA transposons were categories into terminal inverted repeat (TIR), circular dsDNA intermediate (CirdsDNA), DNA polymerase (DP), and circular ssDNA intermediate (rolling circle, RC). Besides, low complexity, satellite, and simple repeat were also included.

To examine the diversity of repeat families, the 459 plant species were divided into 15 clades based on their phylogeny: algae, bryophyte, lycophytes, fern, gymnosperms, ANA (early angiosperms), magnolids, monocots, base eudicots, super rosids, fabids, malvids, super asterids, lamiids, and campanulids (The Angiosperm Phylogeny Group 2016) (Supplementary Table 1b; Supplementary Fig. 2). The abundance and diversity of each repeat type in each clade were characterized.

**Fig. 1:**
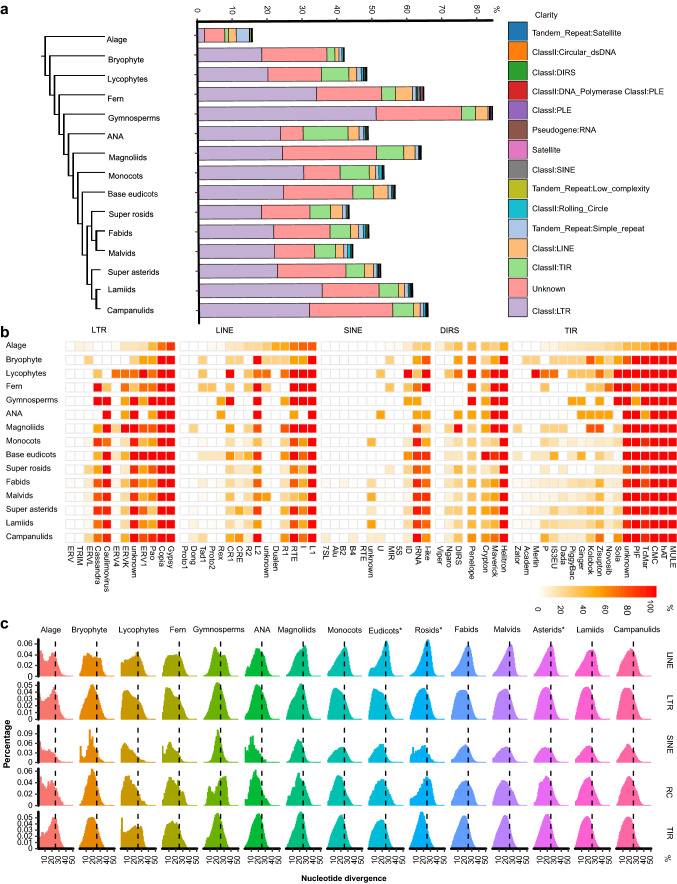
Contribution of repeat to evolution of plant genome. **a** The average percentage of different types of repeats within the genome of 15 groups from green plant kingdom. The left panel displayed the phylogenetic trees of the plant lineages. **b** The percentage of species carrying certain repeat family in each group. **c** Nucleotide diversity of transposons within each plant lineages. The *x*-axial label means the nucleotide diversity percentage of repeat. The vertical dashed line represents the divergence rate of 20%. Eudicots*, base eudicots; Rosids*, super rosids; Asterids*, super asterids

We examined the distribution of different types of repeat sequences in each clade (Supplementary Table 1c). The average percentage of repeats within the genome across all species was 45.49%. The top five abundant types of repeats are LTR, TIR, LINE, Simple repeat, and Rolling Circle, accounting for 21.66%, 5.44%, 2.25%, 1.54%, and 0.59% of the plant genome on average (Fig. 1a; Supplementary Table 2), respectively. Plants from different lineages display distinct proportion of repeat types. In general, the proportion of repeats increased from algae, bryophyte, lycophytes, and fern to gymnosperms (Supplementary Figs. S2, S3, S4, S5, S6, S7, S8, S9, S10, S11, S12, S13, S14, S15). LTRs, as the largest family of plant repeats, might be the major contributor for the increase of total TE population. For algae, the proportions of simple repeats and LINEs exceeded LTRs, which were the highest in all the lineages, suggesting a possible mechanism of controlling the LTR amplification in algae. Ferns showed a higher proportion of SINE transposons. In ANA, TIRs accounted for a prominent proportion of repeats. The diverse compositions of the repeat types among plant species could provide a source for their unique genome evolution trajectory.

To trace back the evolutionary history of plant transposons, we analyzed the presence/absence of types of repeats (Fig. 1b; Supplementary Table 3; Supplementary Figs. S16, S17). We found that most of the repeat families existed in algae, indicating that the common ancestor of algae and land plants had already evolved the fundamental transposon layout of modern green plants (Fig. 1b).

The diversity of transposon nucleotide can reflect the evolution rate of transposon to some extent, we investigate the nucleotide diversity of transposons for each plant linage (Fig. 1c; Supplementary Table 4). In plants, the main nucleotide diversity peaks of the LTR concentrates are at 16%, and the TIR is about 20%, but the LINE concentrates are around 25%, indicating that LINE lack recently replication activity compared to LTR and TIR. The result to some extent explains the genome different between plant and animal: the proportion of LINE in the plant genome is usually less than 5%, but in some animal, LINE is the main repeat content of genome. As the main contributor to plant genome, LTRs were selected to investigate the evolutionary history of amplification (Fig. 1c; Supplementary Table 4). The main peak of algae is 0.2–0.23, which is smaller than the peaks of all of the other species, implying that algae carried ancient LTR groups. LTR amplified more recently in bryophytes compared with algae. The nucleotide diversity of LTR in ferns and lycophytes showed the lowest diversity across all the land plants. The amplification of LTR in the genome of gymnosperms leads to a large genome, and the results show that the degree of divergence of LTR is high, indicating that it has no recent activity, which is consistent with the results of Norway spruce. The nucleotide diversity of ANA and magnoliids is similar as that of gymnosperms. Monocots and base eudicots displayed a more recent amplification. For dicots, the nucleotide diversity was broad, indicating several rounds of amplification of LTRs. According to the divergence of LTRs, we also estimated the amplification time of LTRs along the plant, with the main detected amplicon that can be traced back to 1–4 Mya ago (Supplementary Fig. S18). This indicates that the LTRs amplify independently in each linage, playing important roles in the evolution of genome size and environmental adaptability.

To elucidate the contribution of repeats to genes, we calculate the frequencies of repeat sequences at different sites around genes. We found that LTRs, LINEs, SINEs, and DNA transposons display decrease of frequency from 1 kb upstream to 1 kb downstream of transcriptional start site genes (Supplementary Fig. S19; Supplementary Table 5), indicating that plants tend to suppress transposon insertions around gene transcription start sites (TSS); the transposons located near the gene might impact the expression and function of gene. The common feature of LTRs and LINEs near the gene is that there is an inflection point which falls sharply from 2 kb upstream of the TSS to the lowest frequency at 1 kb within genes TSS (Supplementary Figs. S19, S20, S21). It then continues to rise within the gene to a distribution of 10 k. The frequency of 10 kb within gene is close to or even higher than the frequency at 10 kb upstream of the TSS. Similarly, the frequency of SINE and DNA transposons from 1 kb upstream of the TSS to 1 kb within the gene decreases, in gene internal frequency rises; but then, the frequency shows a downward or stable trend within gene (Supplementary Figs. S19, S22), which is different from LTRs and LINEs. The result implies that different transposon families might adopt specific integration strategies and occupy different “niches” of genome (Zhang et al. 2020). Unlike TEs, the frequency of simple repeats near TSS is opposite. Simple repeats increase from 4.5 kb upstream of TTS to 0.5 kb within the gene where it reaches the highest frequency (Supplementary Fig. S19), which is similar to the results of before study (Huda et al. 2009). Therefore, one can speculate that the high frequency of simple repeats around the gene provides a certain fault tolerance rate for the stability of gene transcription.

In summary, we re-annotated repeats of 459 plant species and characterized the abundance, presence/absence, and nucleotide diversity of the repeat types for 15 plant taxonomic groups. The frequency of repeats along the gene models showed unique patterns for different repeat types. Our work supplies a new resource for the future study of repeat sequences and will be helpful to plant genome structure annotation.

## Supplementary Information

Below is the link to the electronic supplementary material.

Supplementary file1 (PDF 2501 kb)

Supplementary file2 (XLSX 453 kb)

Supplementary file3 (XLSX 335 kb)

Supplementary file4 (XLSX 1086 kb)

Supplementary file5 (XLS 137 kb)

Supplementary file6 (XLS 43 kb)

## Data Availability

The datasets generated and/or analyzed during the current study are available in the website: http://www.plantrep.cn/.

## Code availability

The code used during the current study is available from the corresponding author on reasonable request.
