# Supplementary material for: PlantRep: a database of plant repetitive elements
Source: Plant Cell Rep. 2022 Jan 3;41(4):1163–6. doi: 10.1007/s00299-021-02817-y (PMC9035001; doi:10.1007/s00299-021-02817-y)
Supplement: Supplementary file 1 — Supplementary file1 (PDF 2501 kb) [file 299_2021_2817_MOESM1_ESM.pdf]

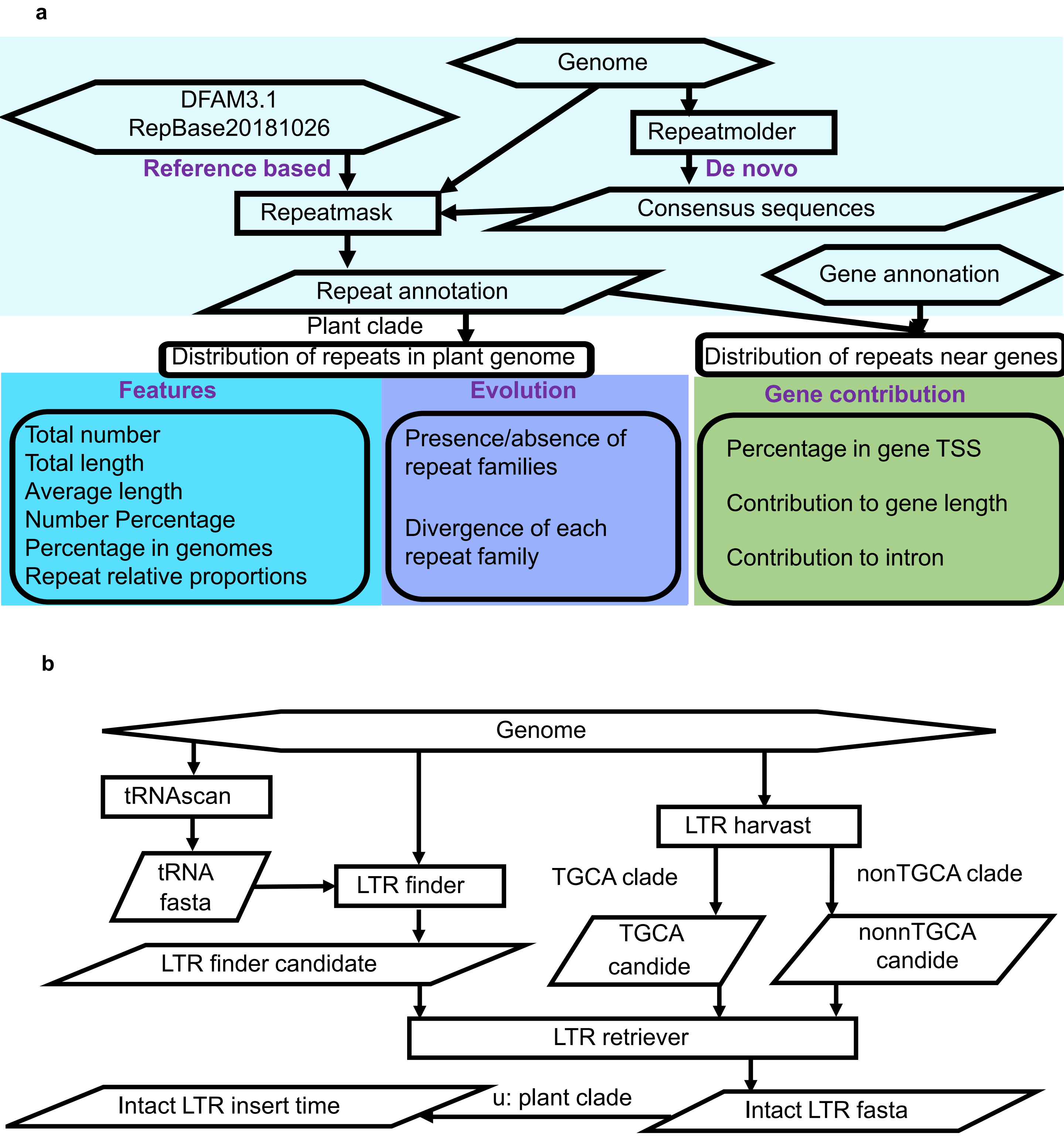

Supplementary Fig S1. Annotation and evolutionary analysis. (a)The pipeline of repeat annotation, statistics and evolutionary analysis. (b) The pipeline to calculate intact LTR insert time.

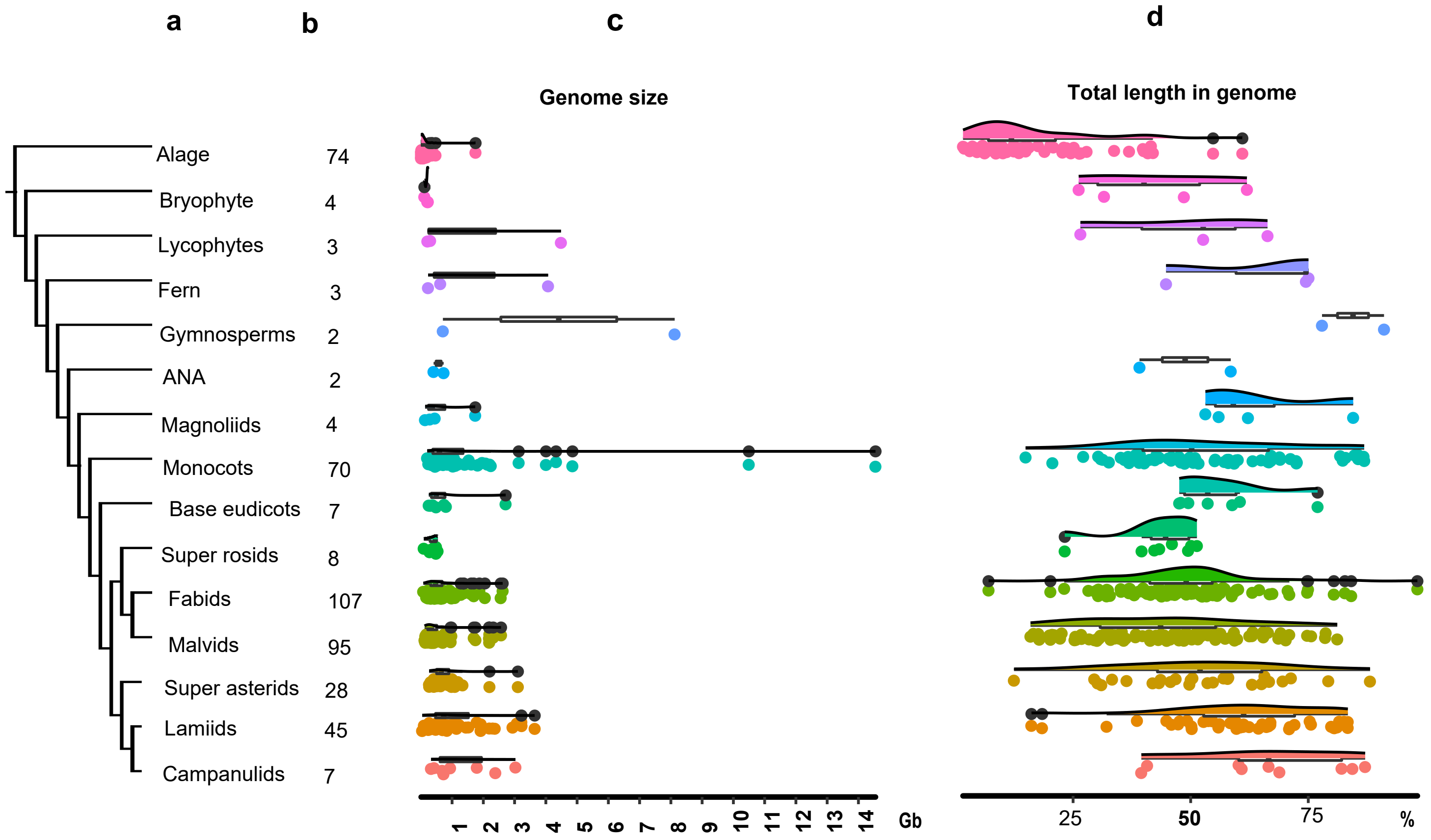

Supplementary Fig S2. Distribution of repeats in different plant genomes. (a) The phylogenetic tree of 15 plant groups from green plant. (b)The number of genomes we used for each groups. (c)The distribution of plant genome size. (d)Percentage of total length of repeats in each plant genome.

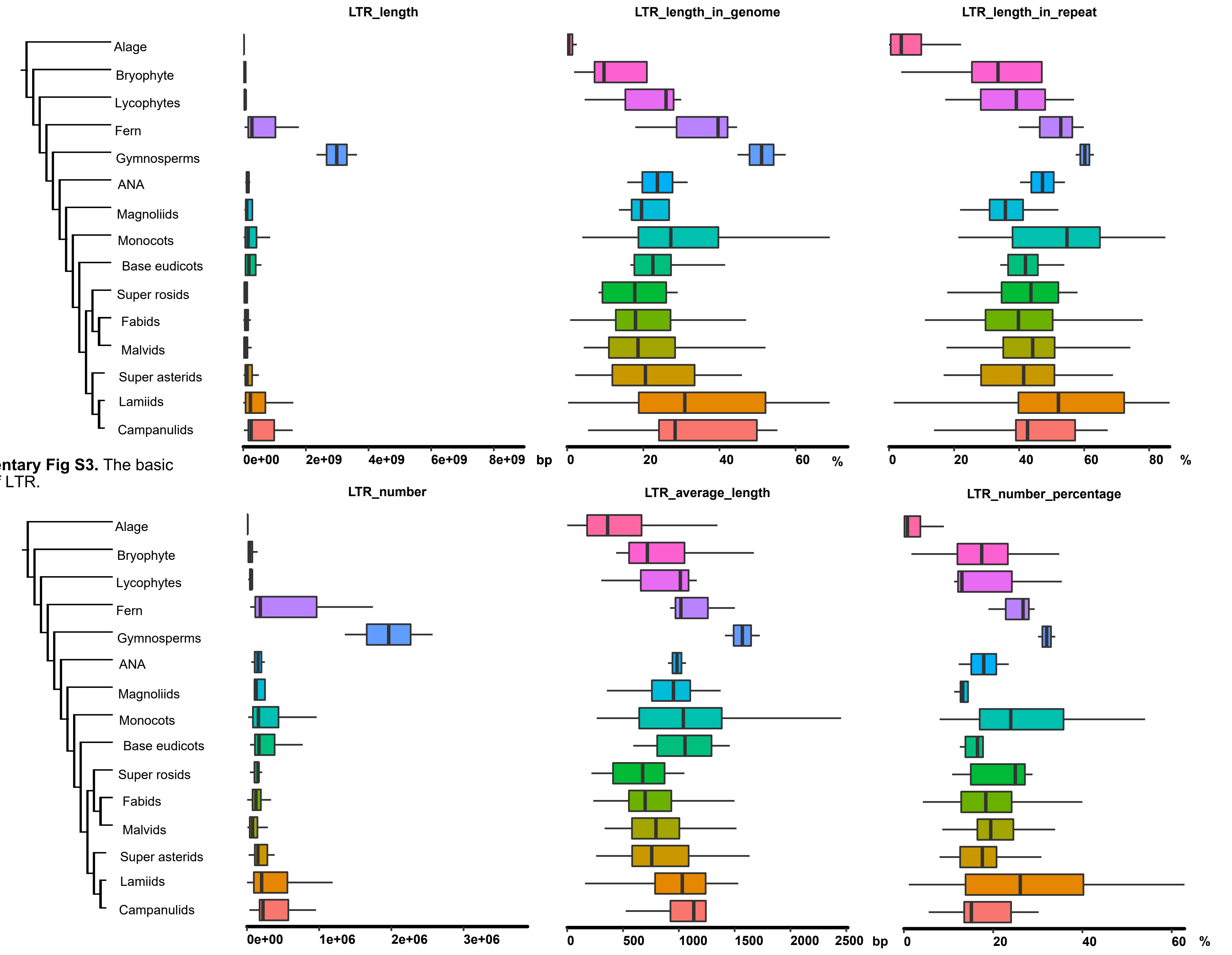

Supplementary Fig S3. The basic features of LTR.

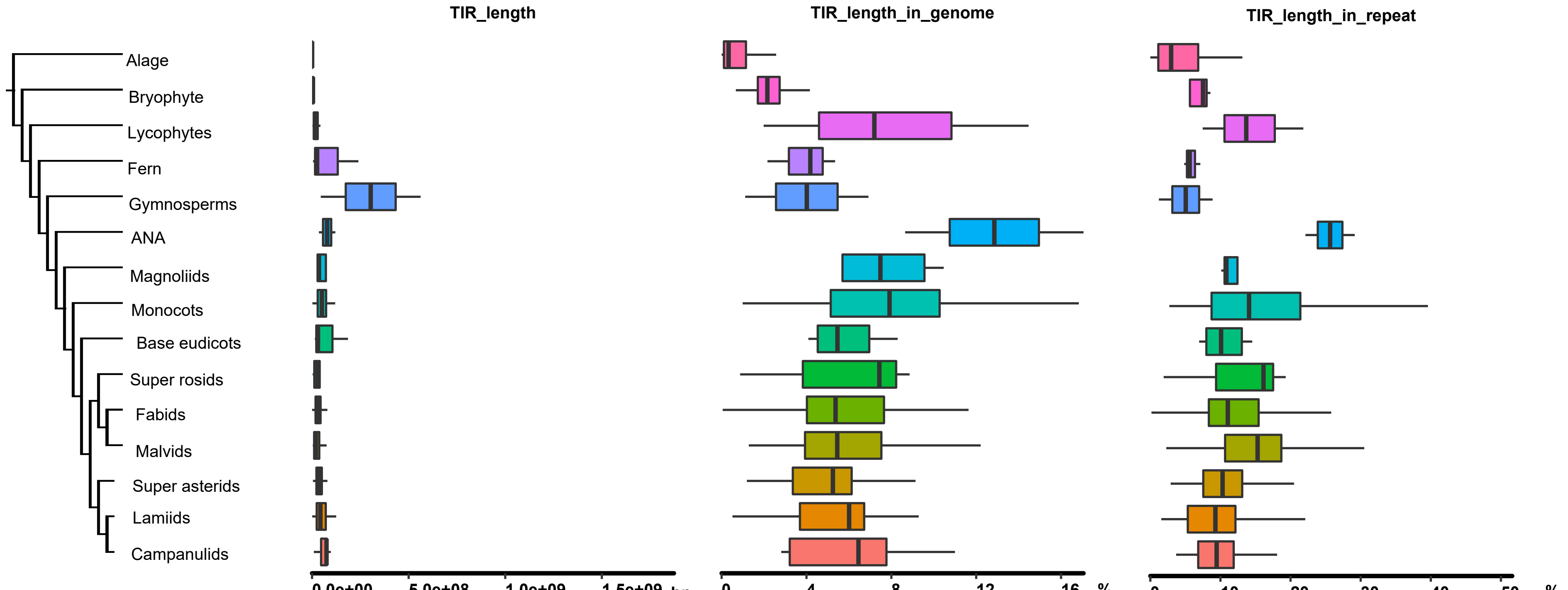

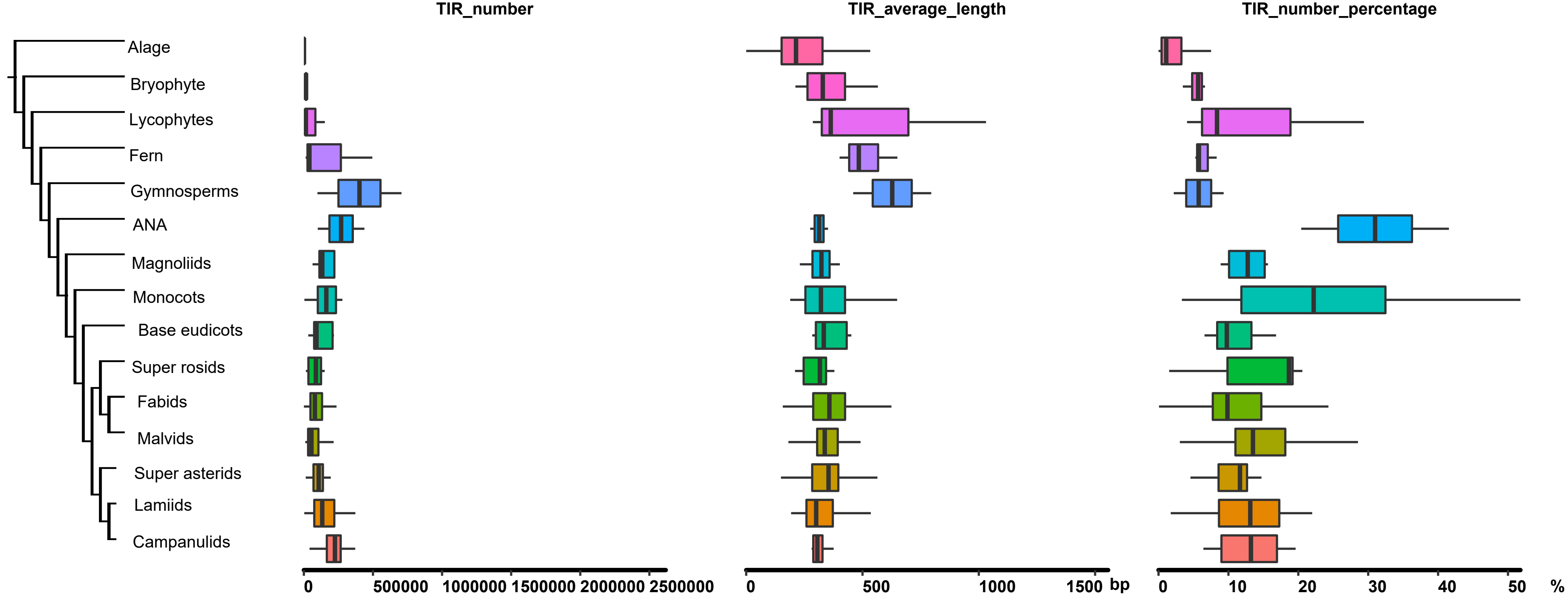

Supplementary Fig S4. The basic features of TIR.

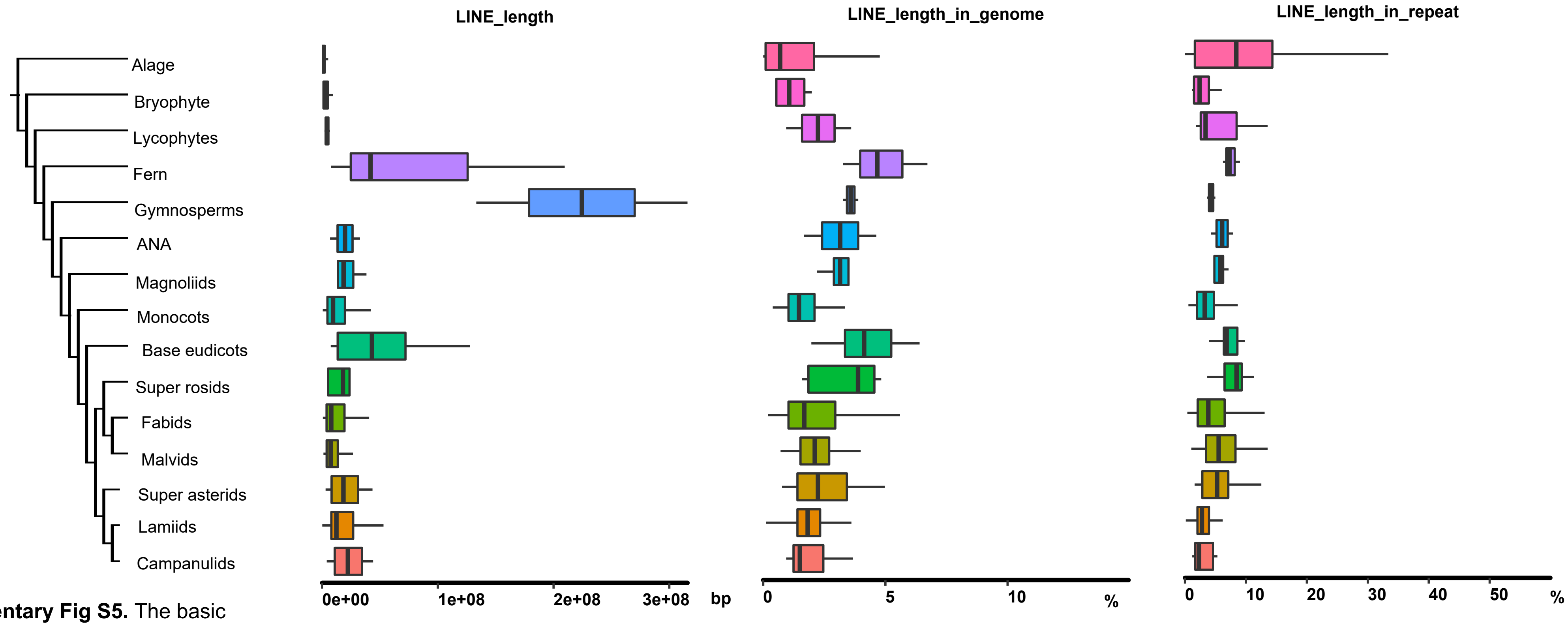

Supplementary Fig S5. The basic features of LINE.

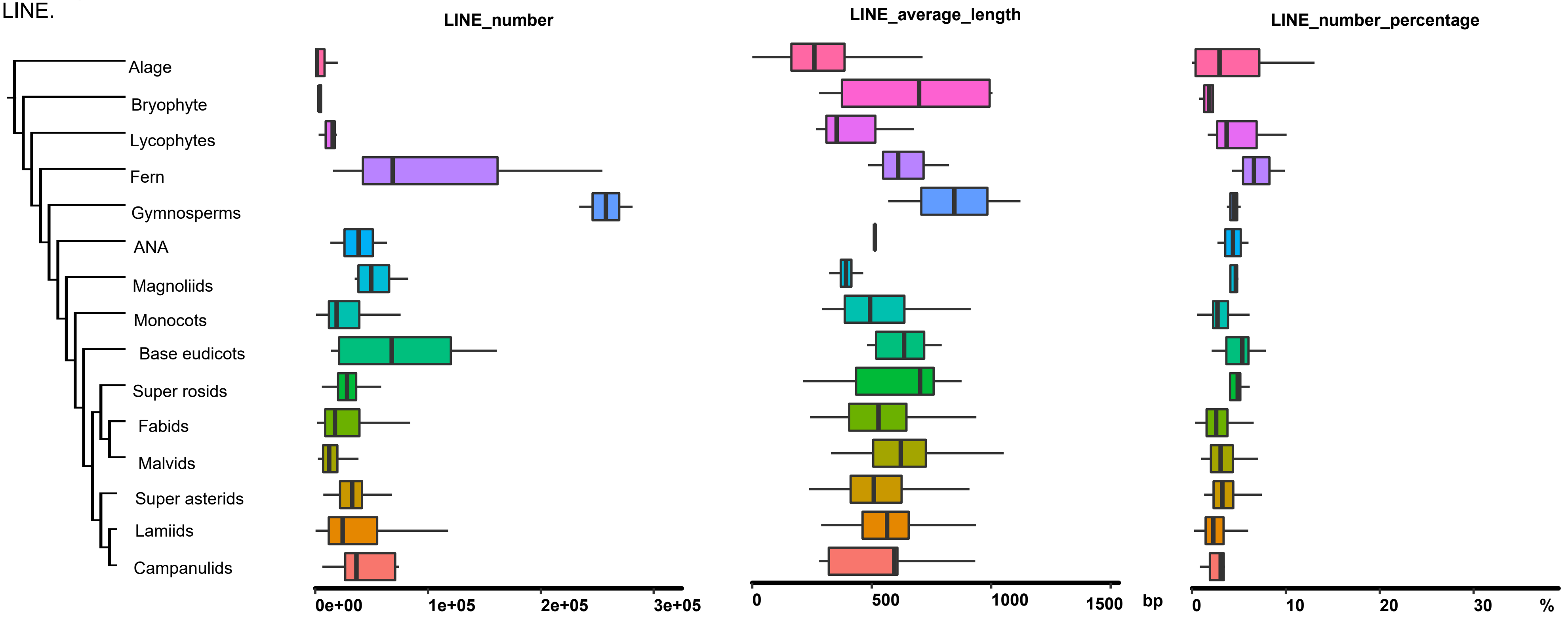

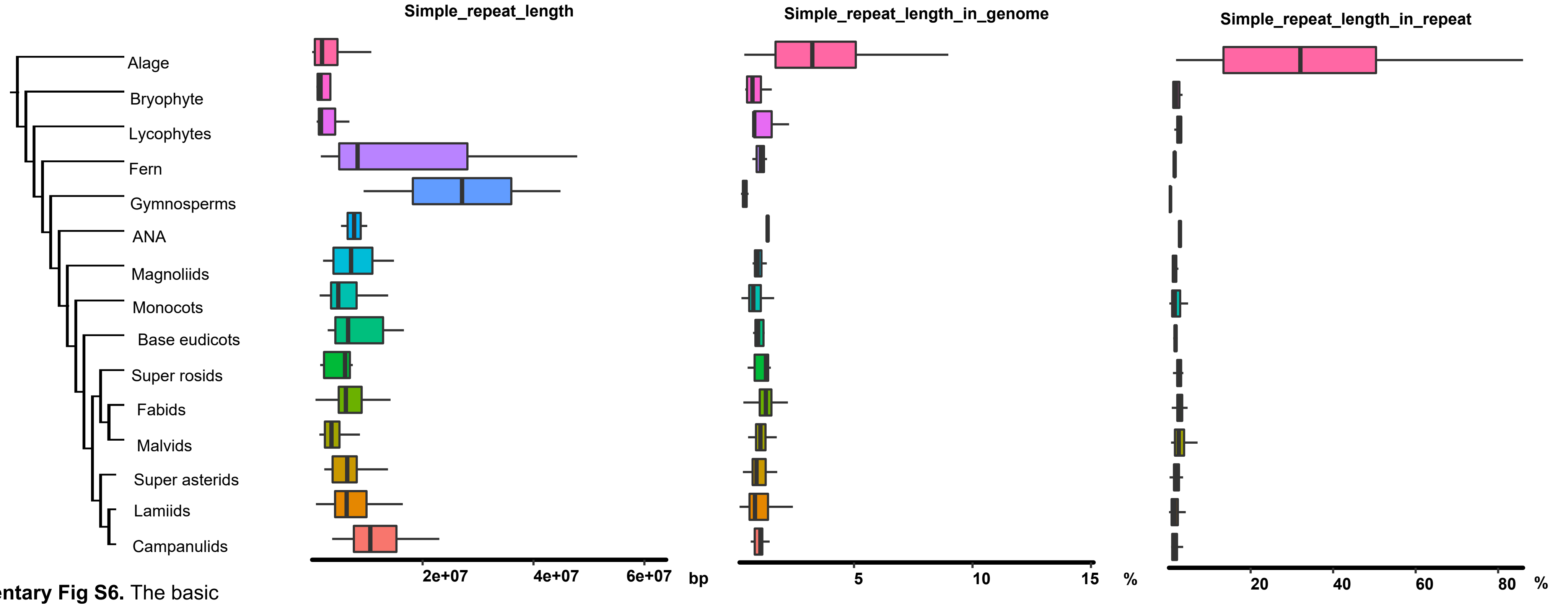

Supplementary Fig S6. The basic features of Simple repeat.

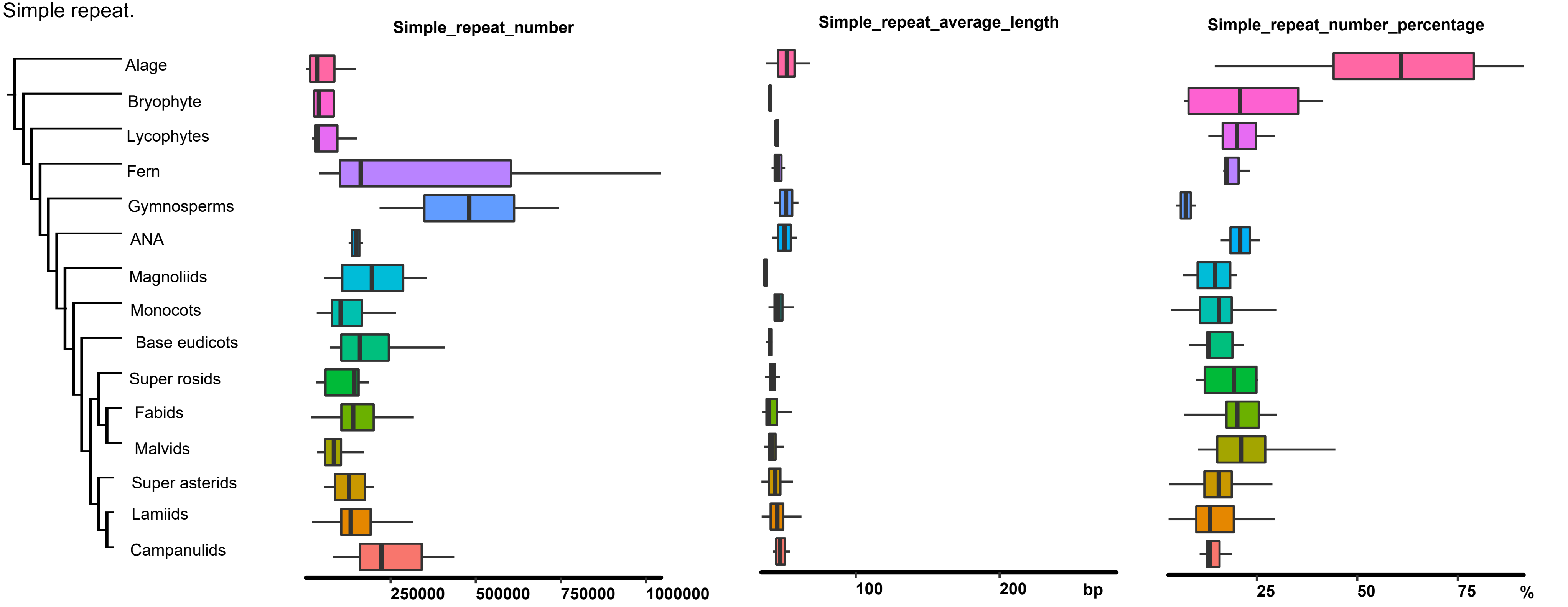

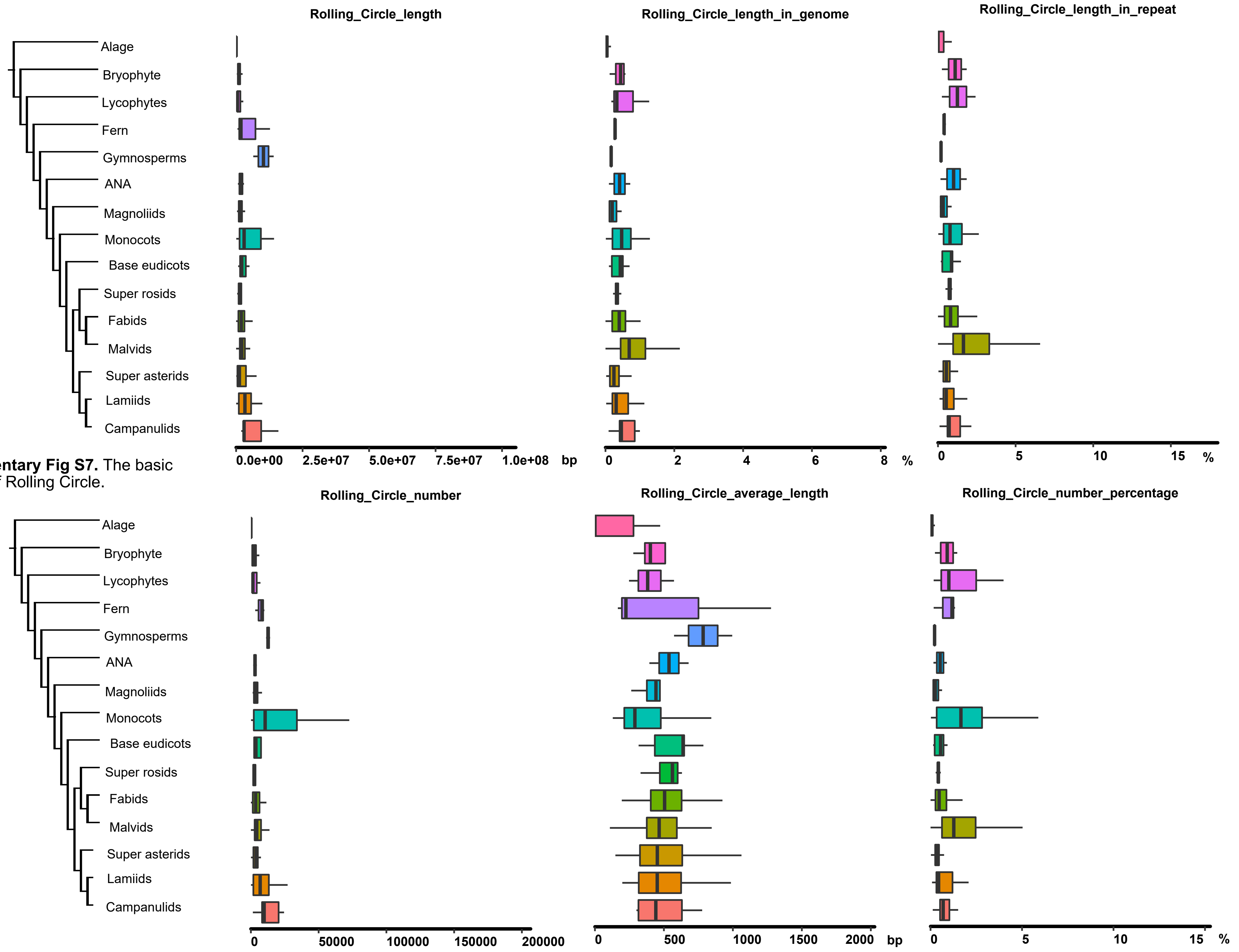

Supplementary Fig S7. The basic features of Rolling Circle.

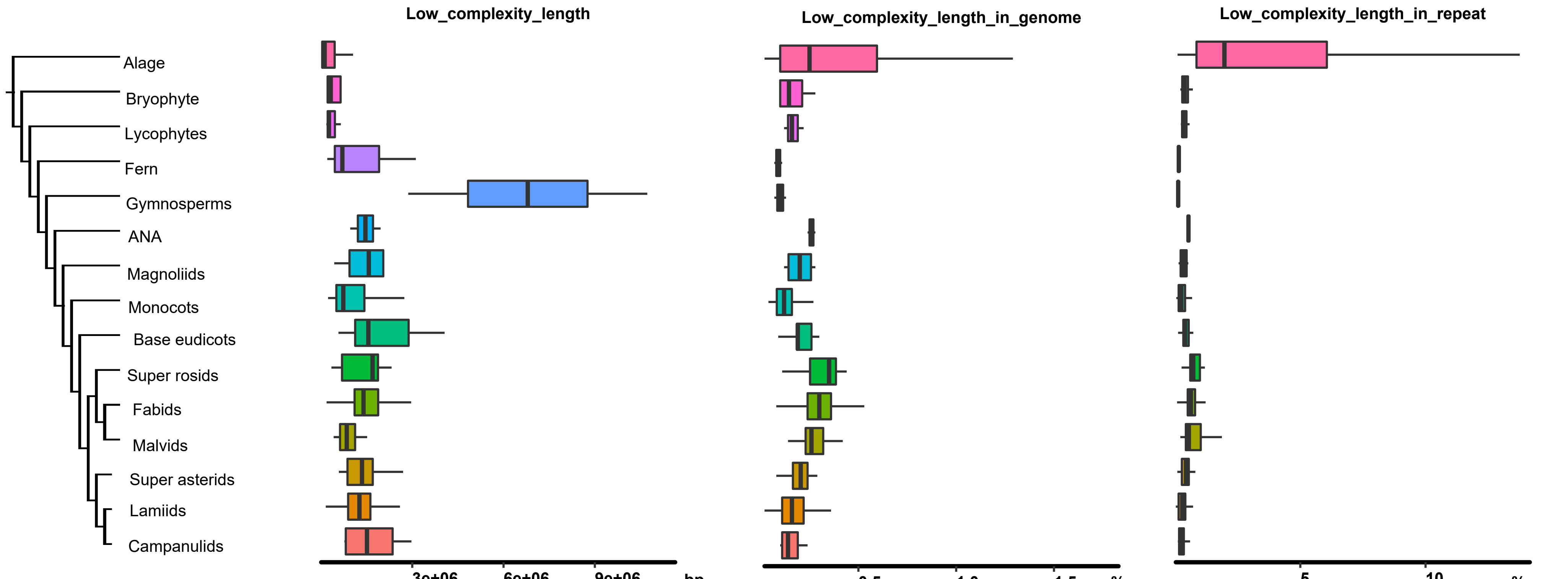

Supplementary Fig S8. The basic features of Low complexity.

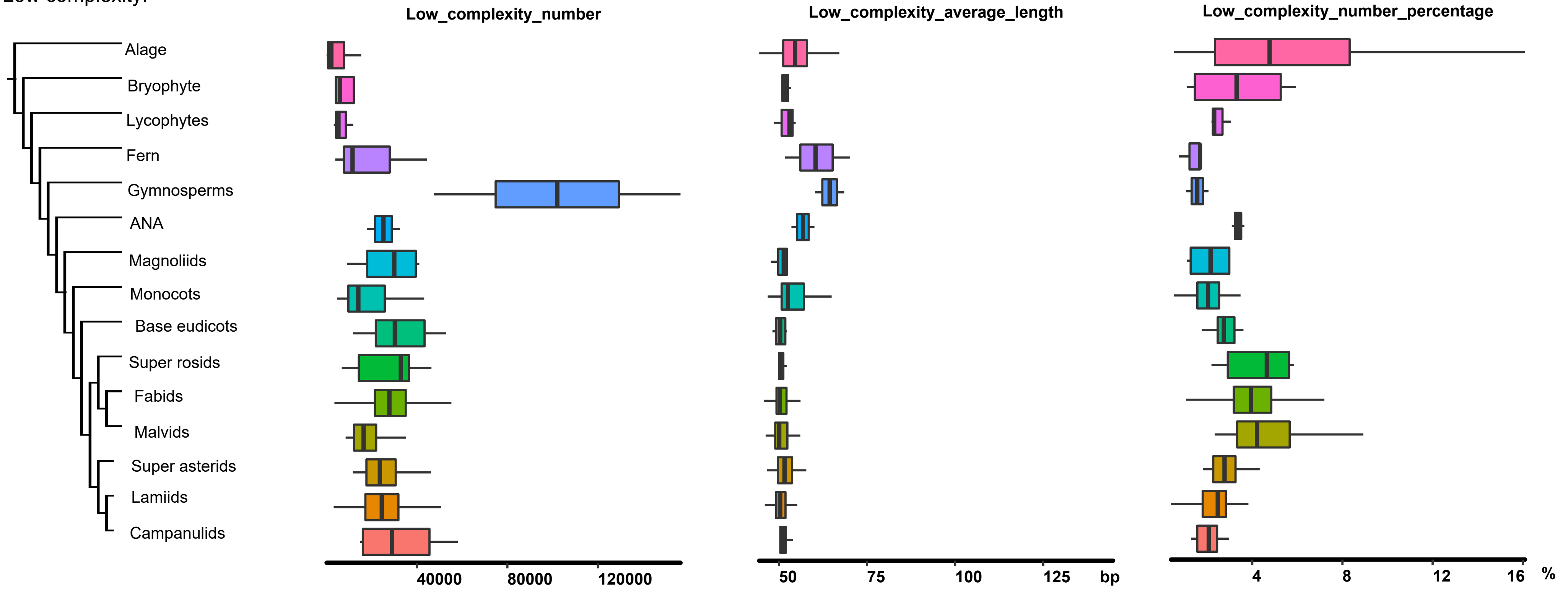

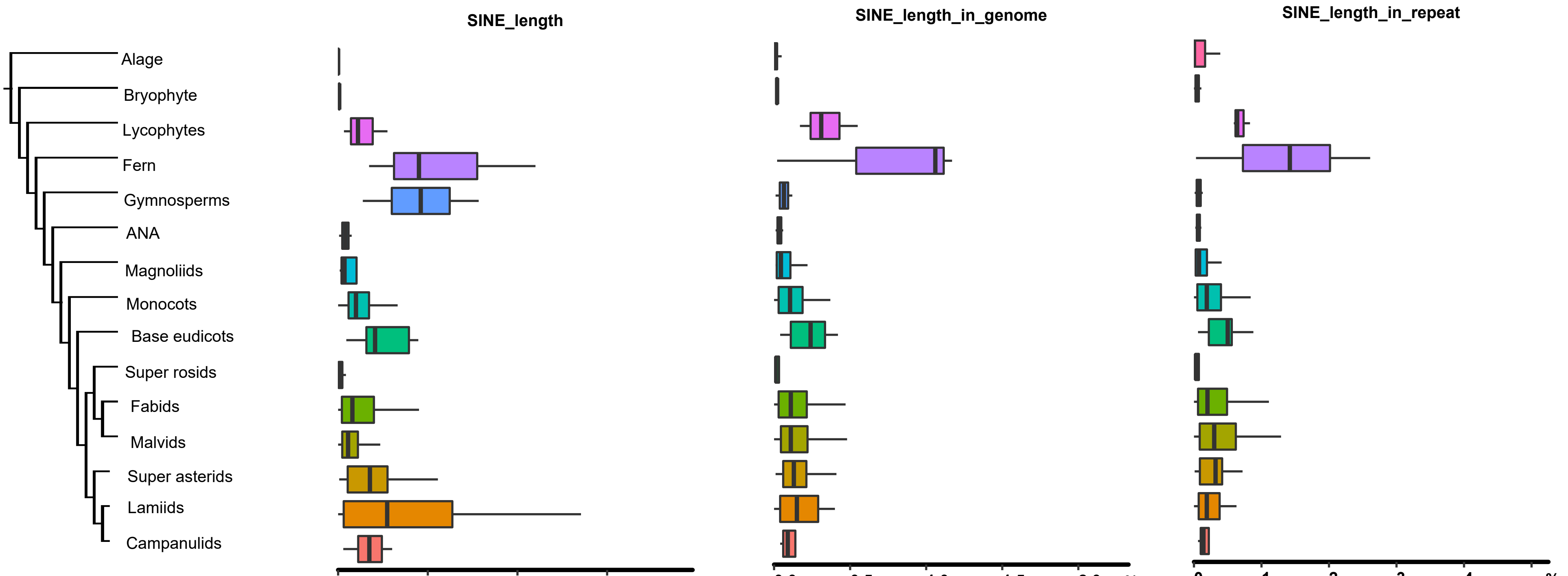

Supplementary Fig S9. The basic features of SINE.

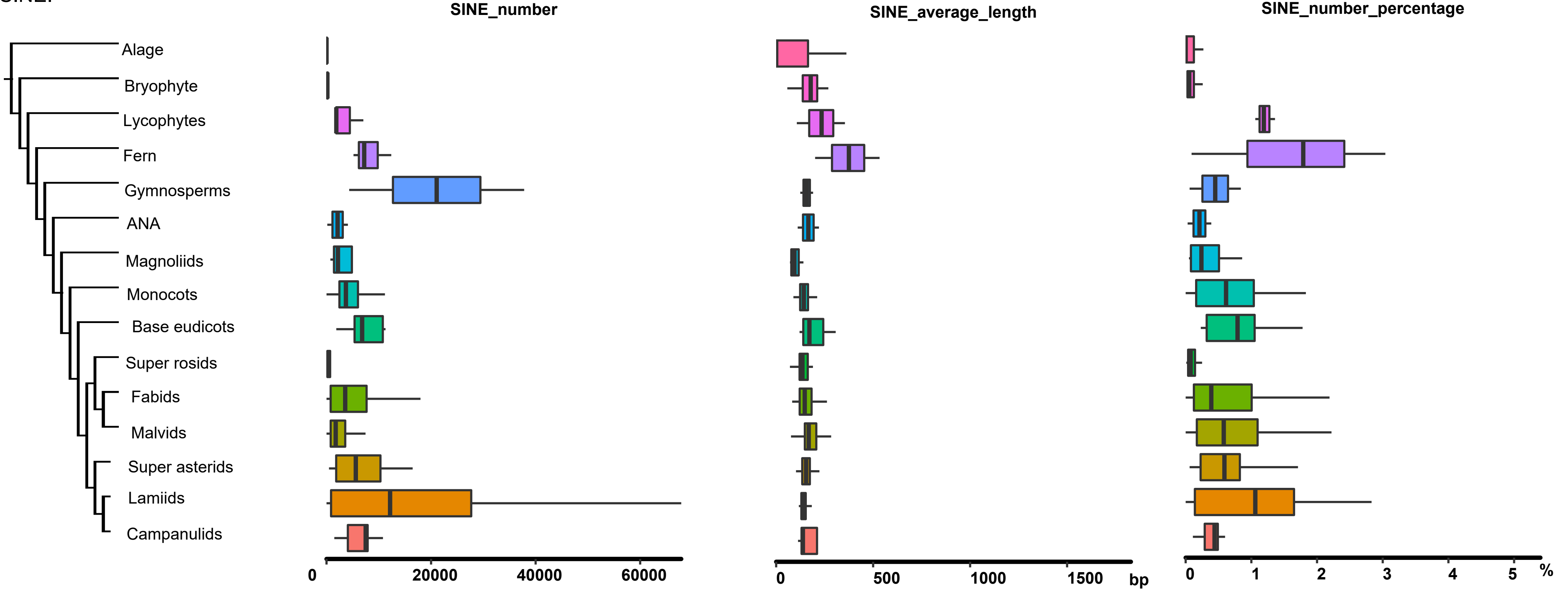

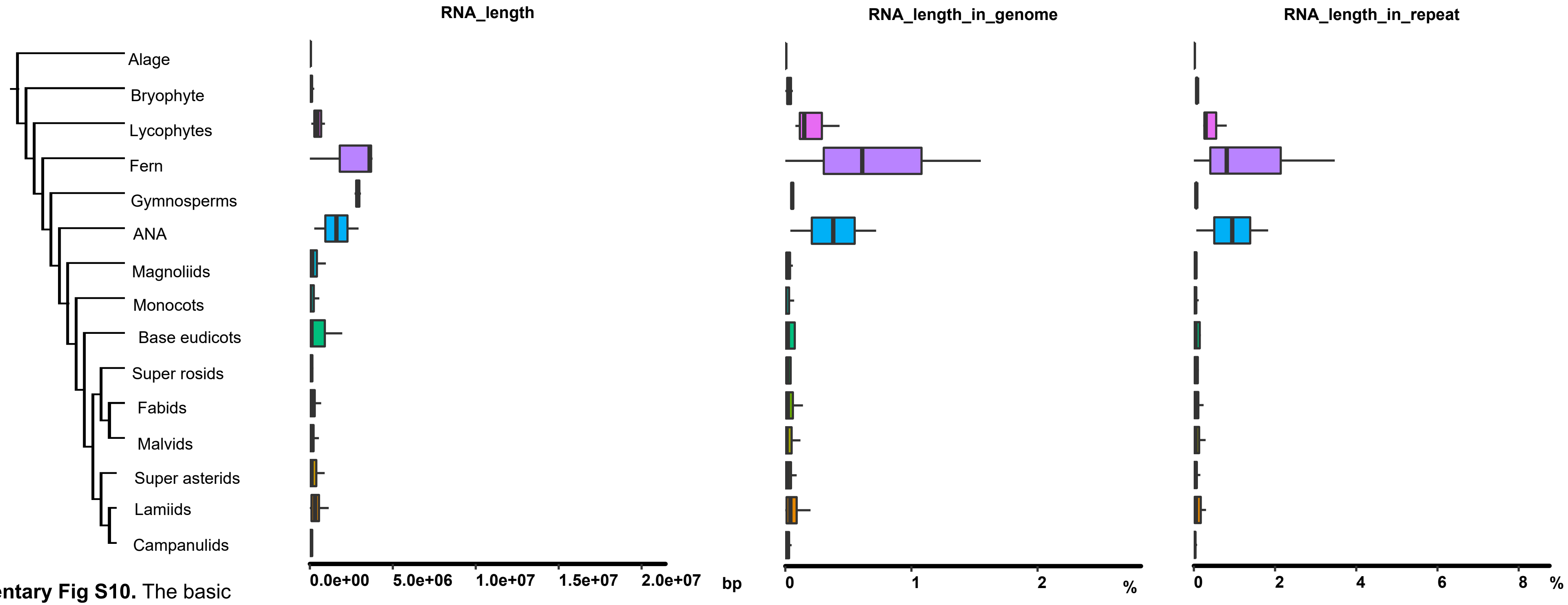

Supplementary Fig S10. The basic features of RNA.

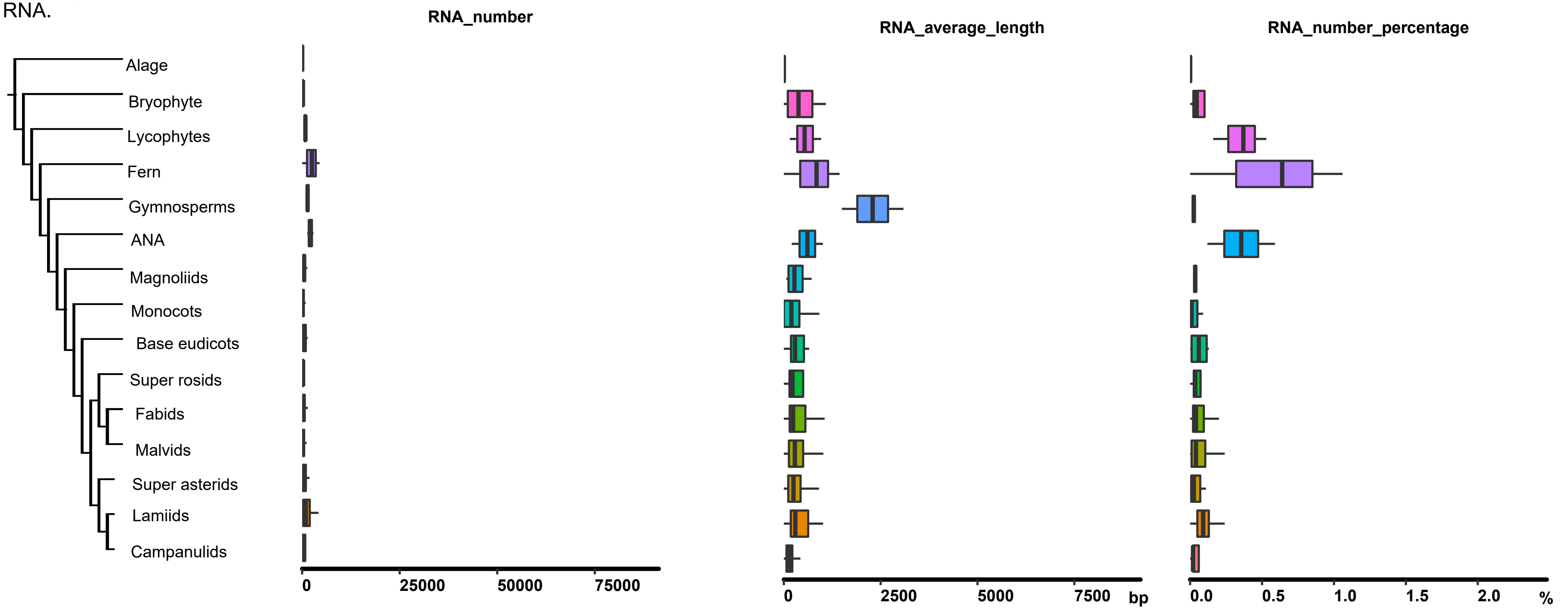

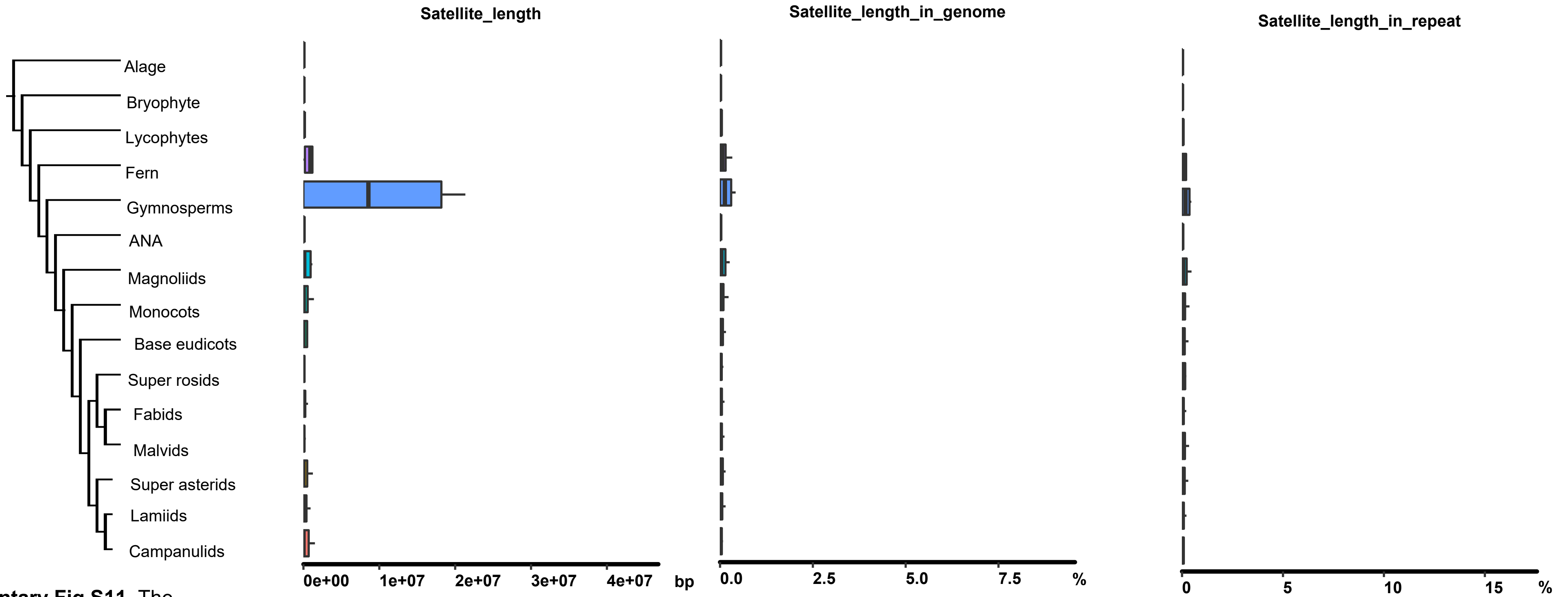

Supplementary Fig S11. The basic features of Satellite.

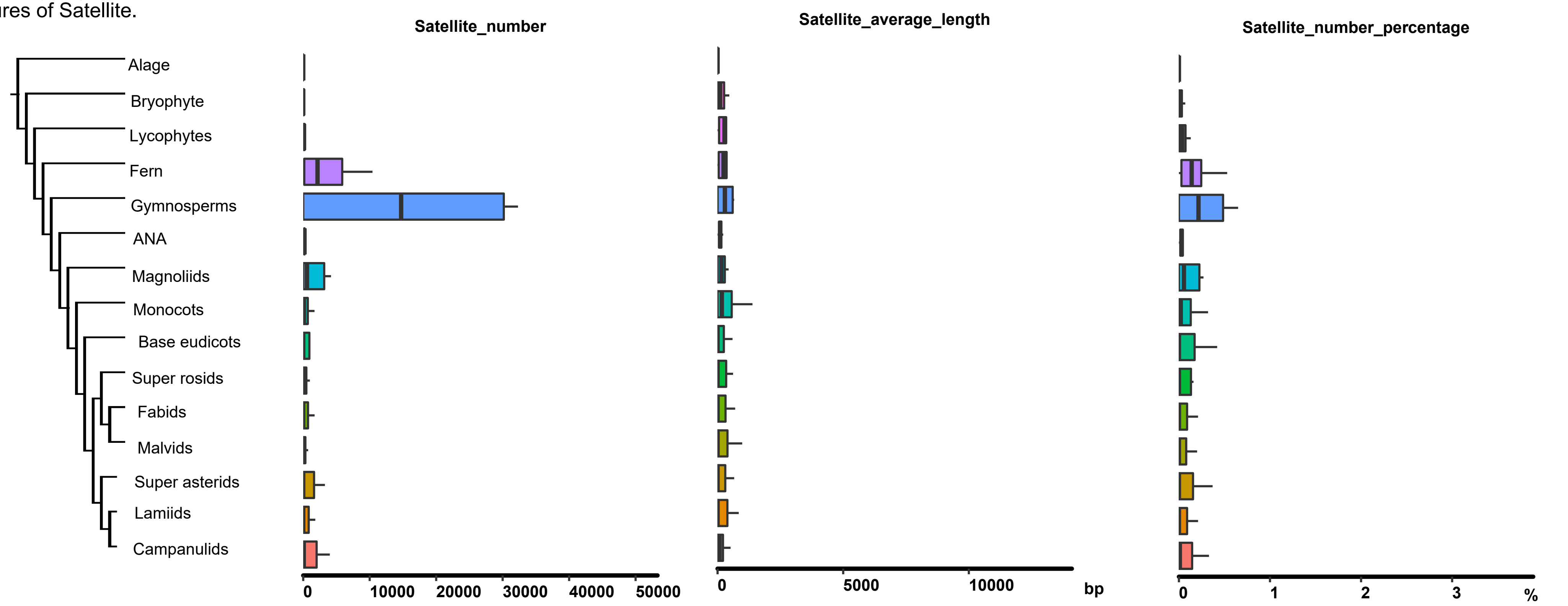

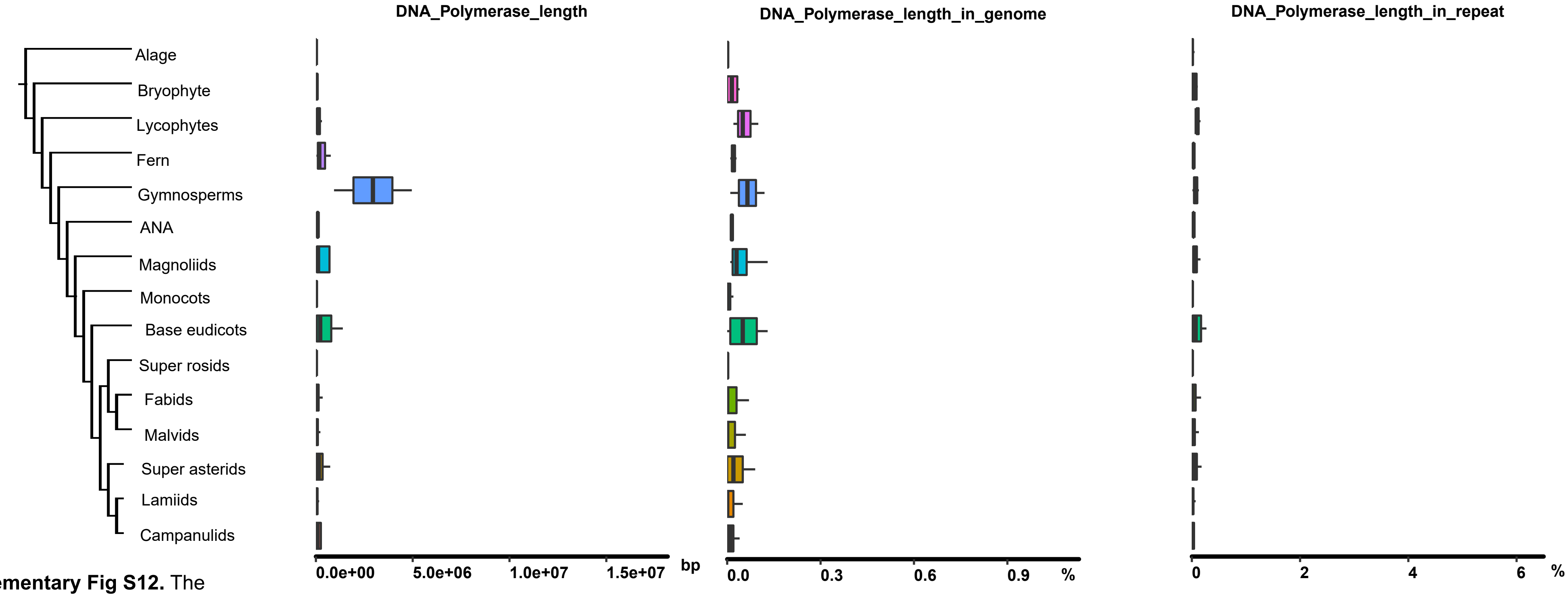

Supplementary Fig S12. The basic features of DNA Polymerase.

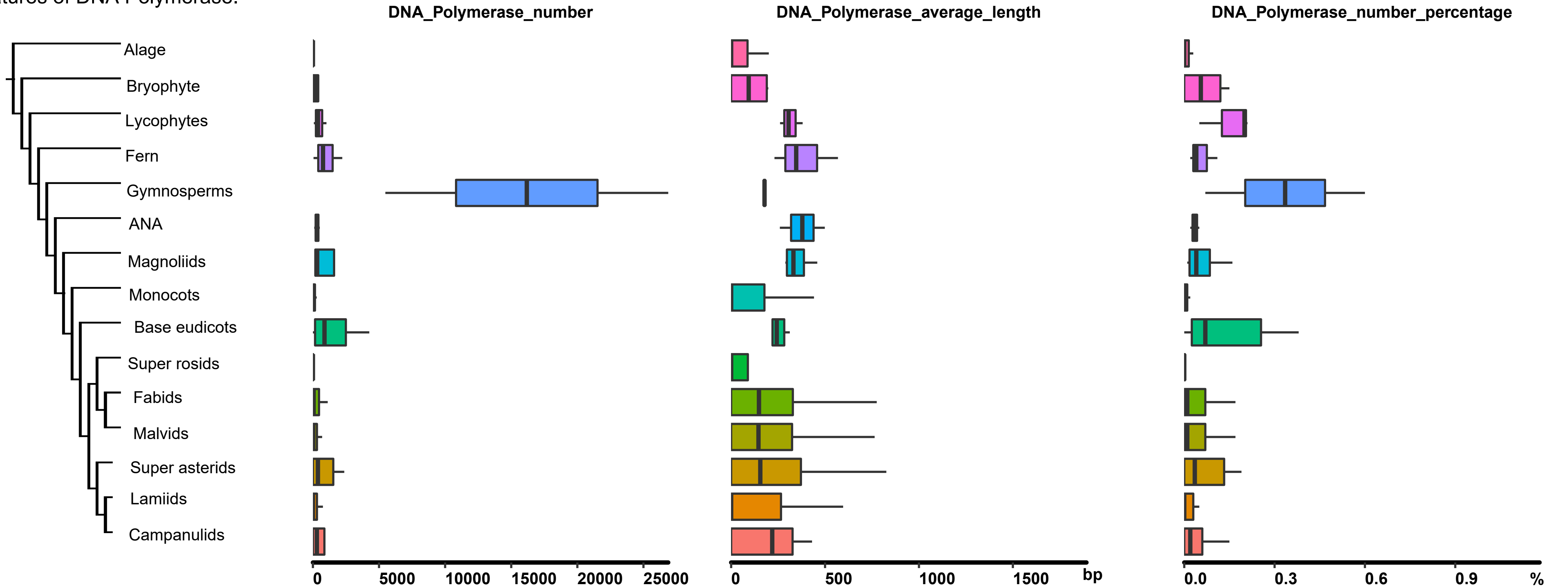

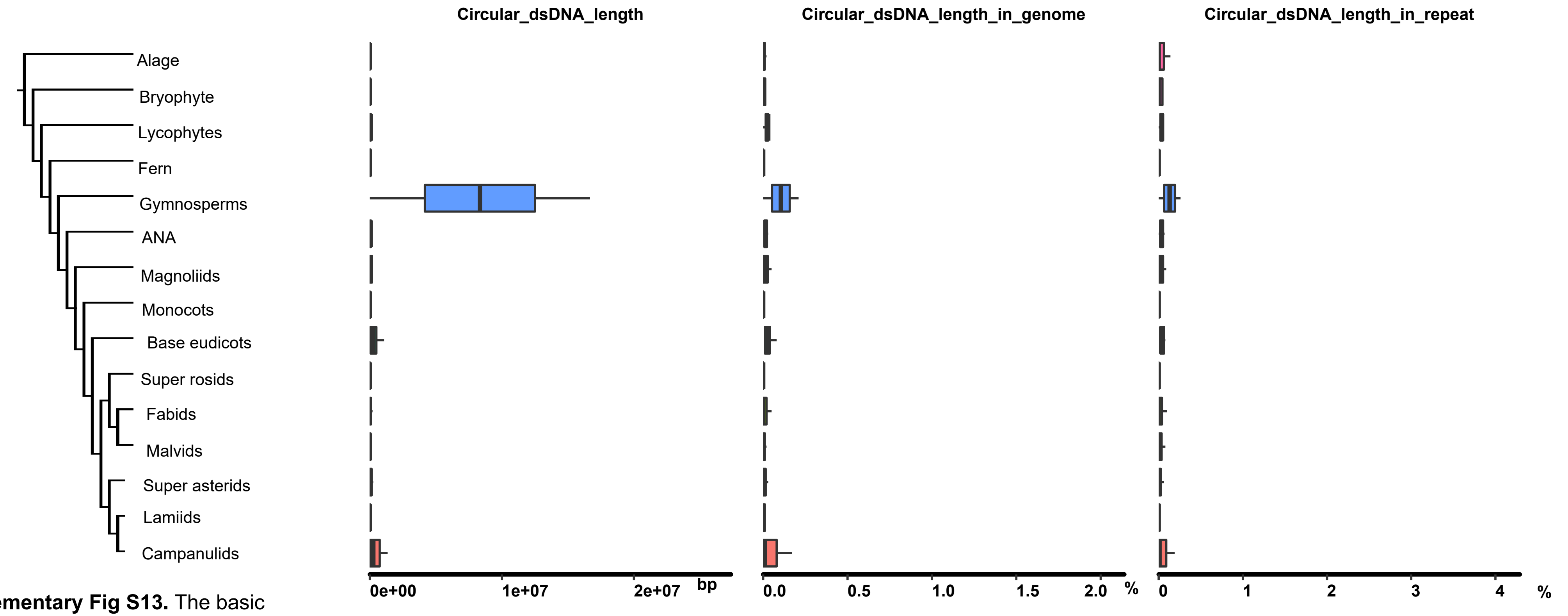

Supplementary Fig S13. The basic features of Circular dsDNA.

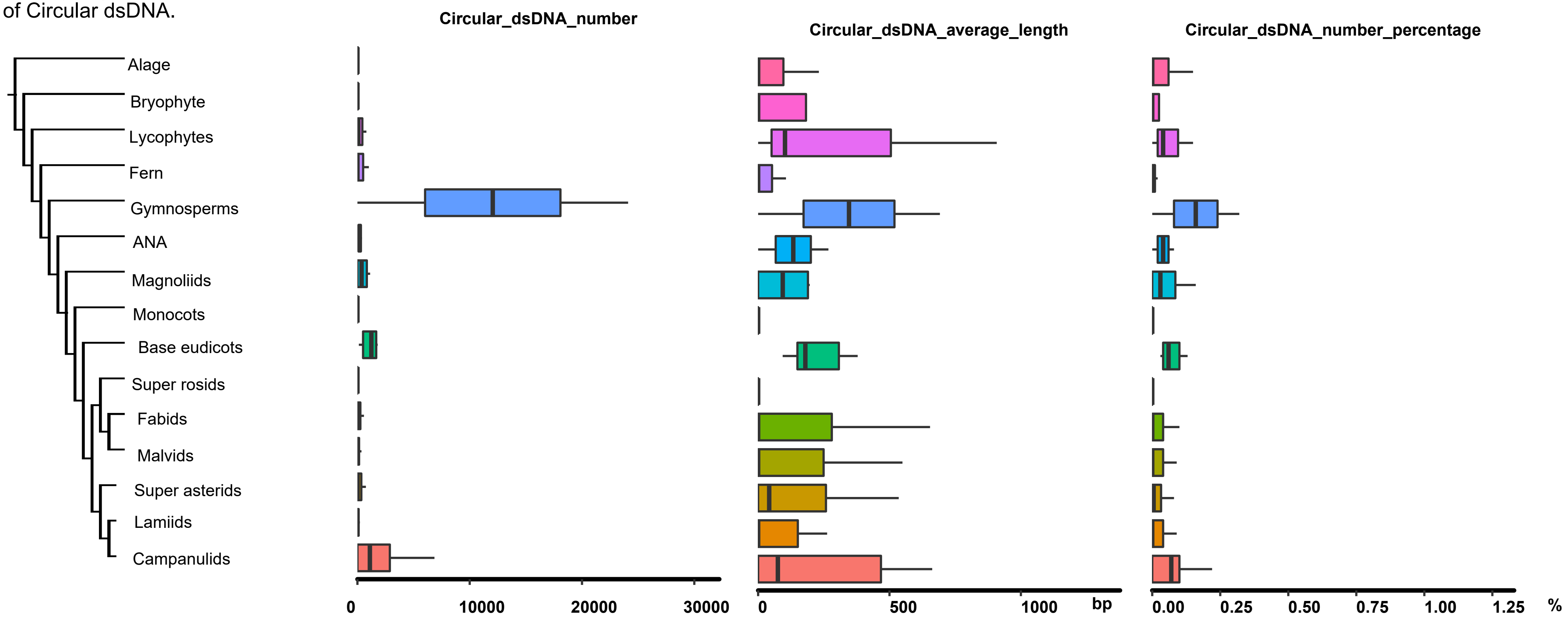

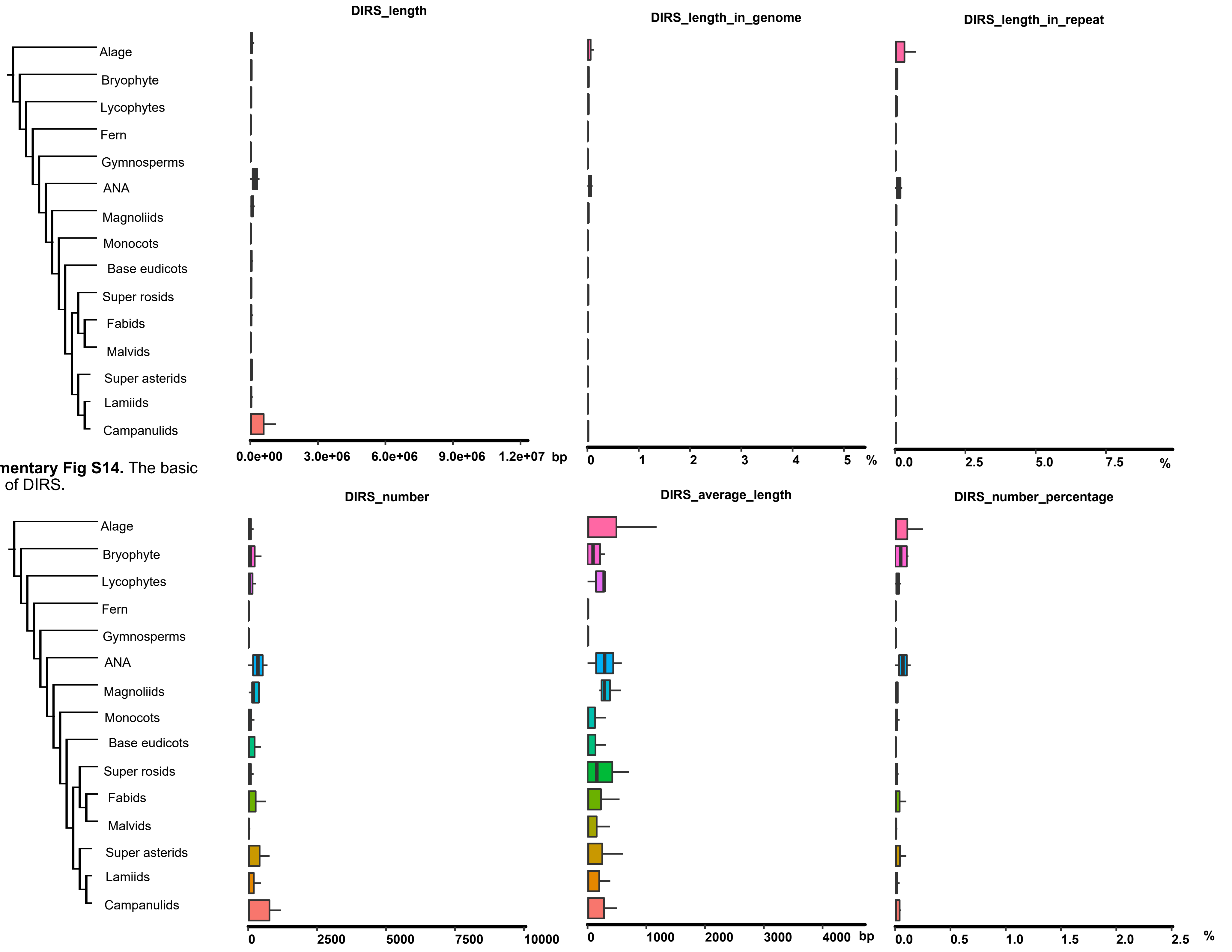

Supplementary Fig S14. The basic features of DIRS.

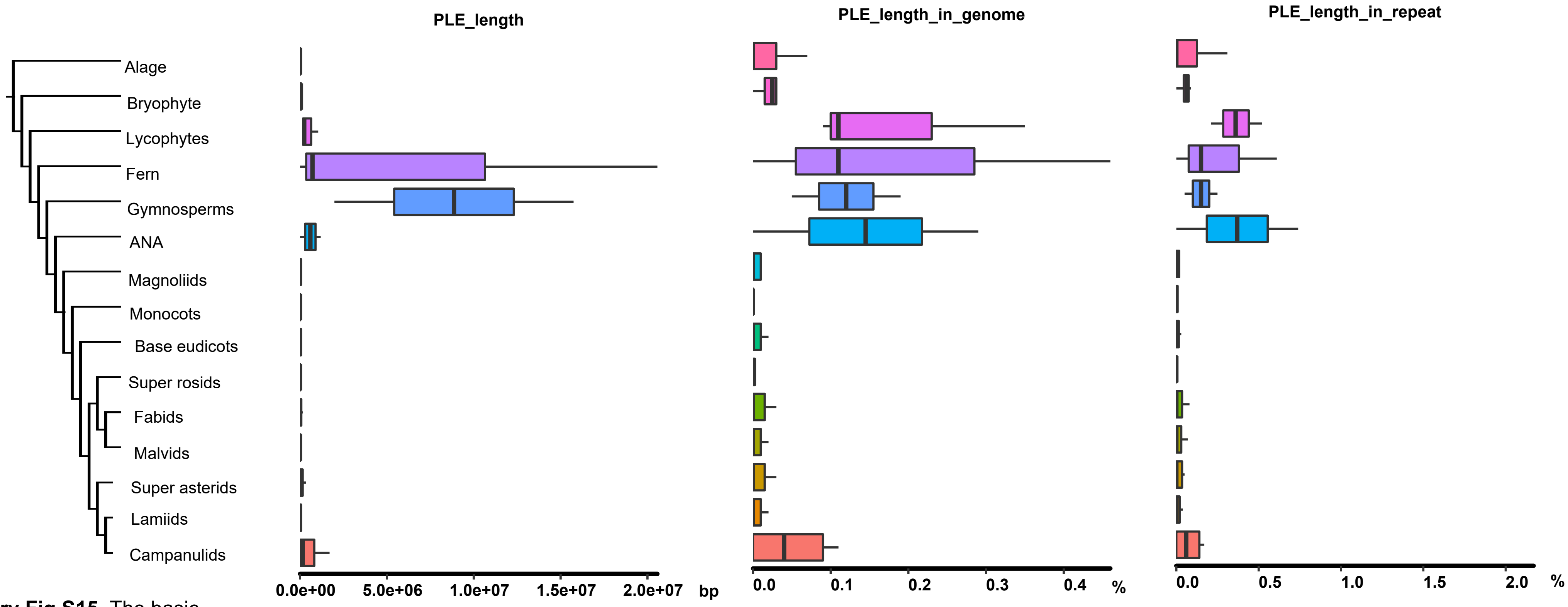

Supplementary Fig S15. The basic features of PLE.

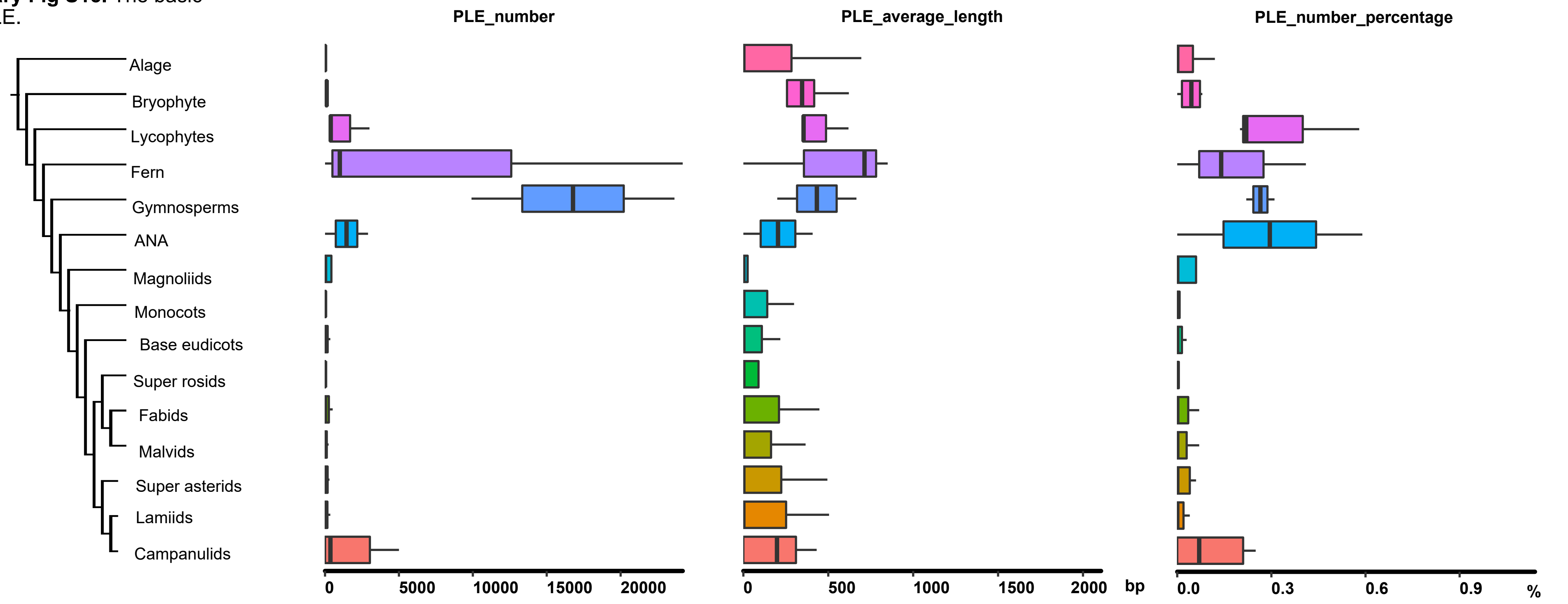

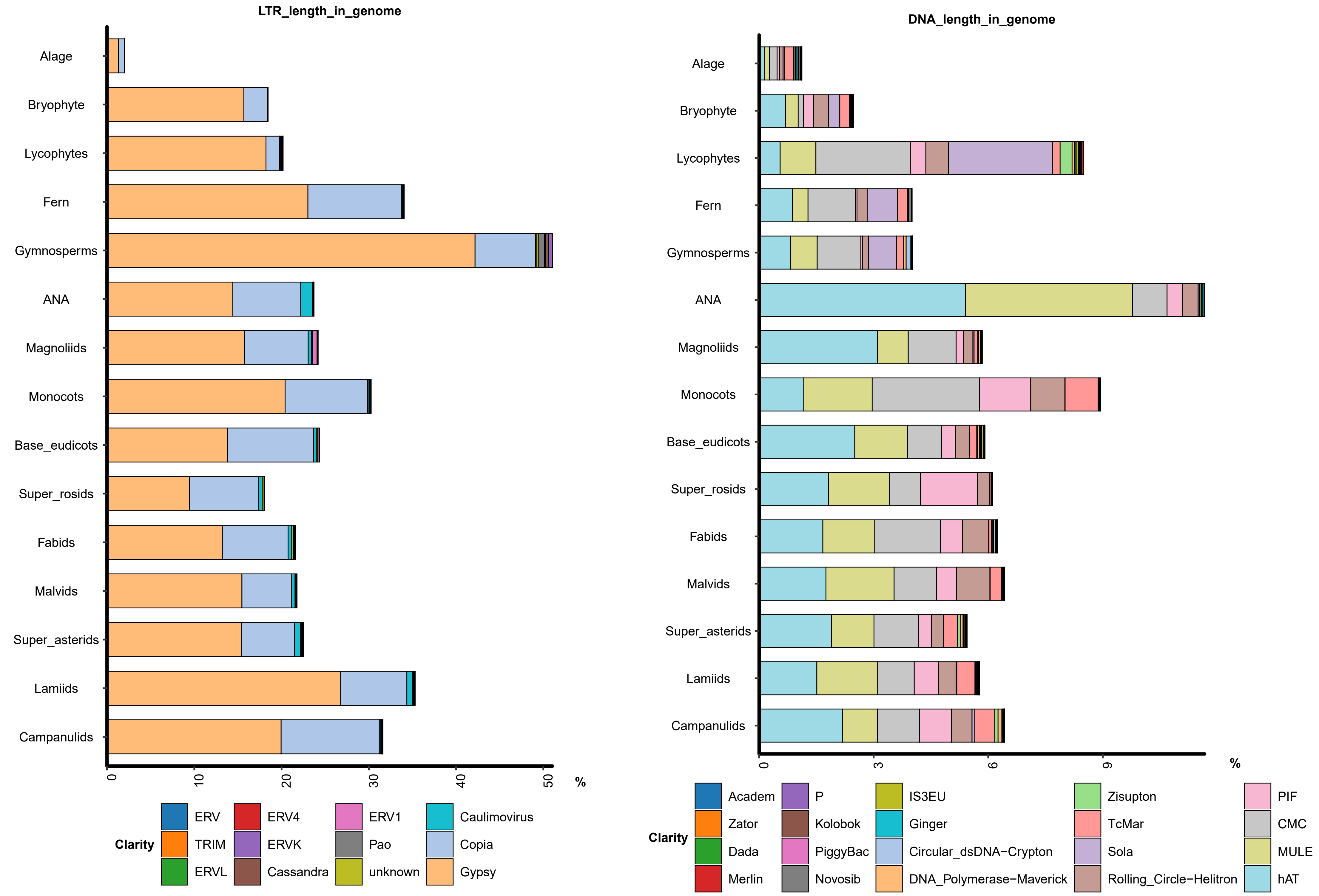

Supplementary Fig S16. (a)Proportion of the length of LTR superfamily in plant genome. (b)Proportion of the length of DNA transposon superfamily in plant genome.

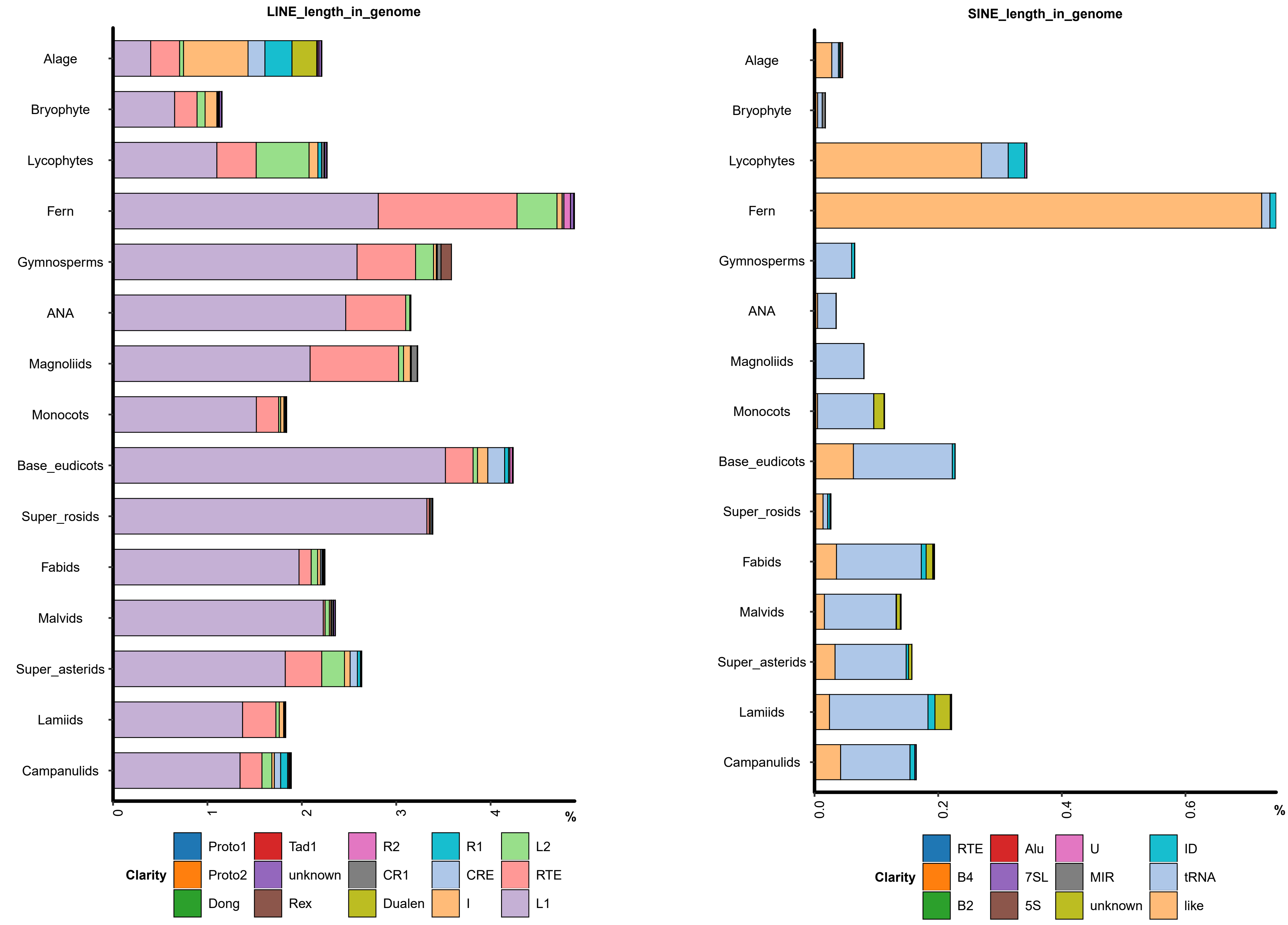

Supplementary Fig S17. (a)Proportion of the length of LINE superfamily in plant genome. (b)Proportion of the length of SINE superfamily in plant genome.

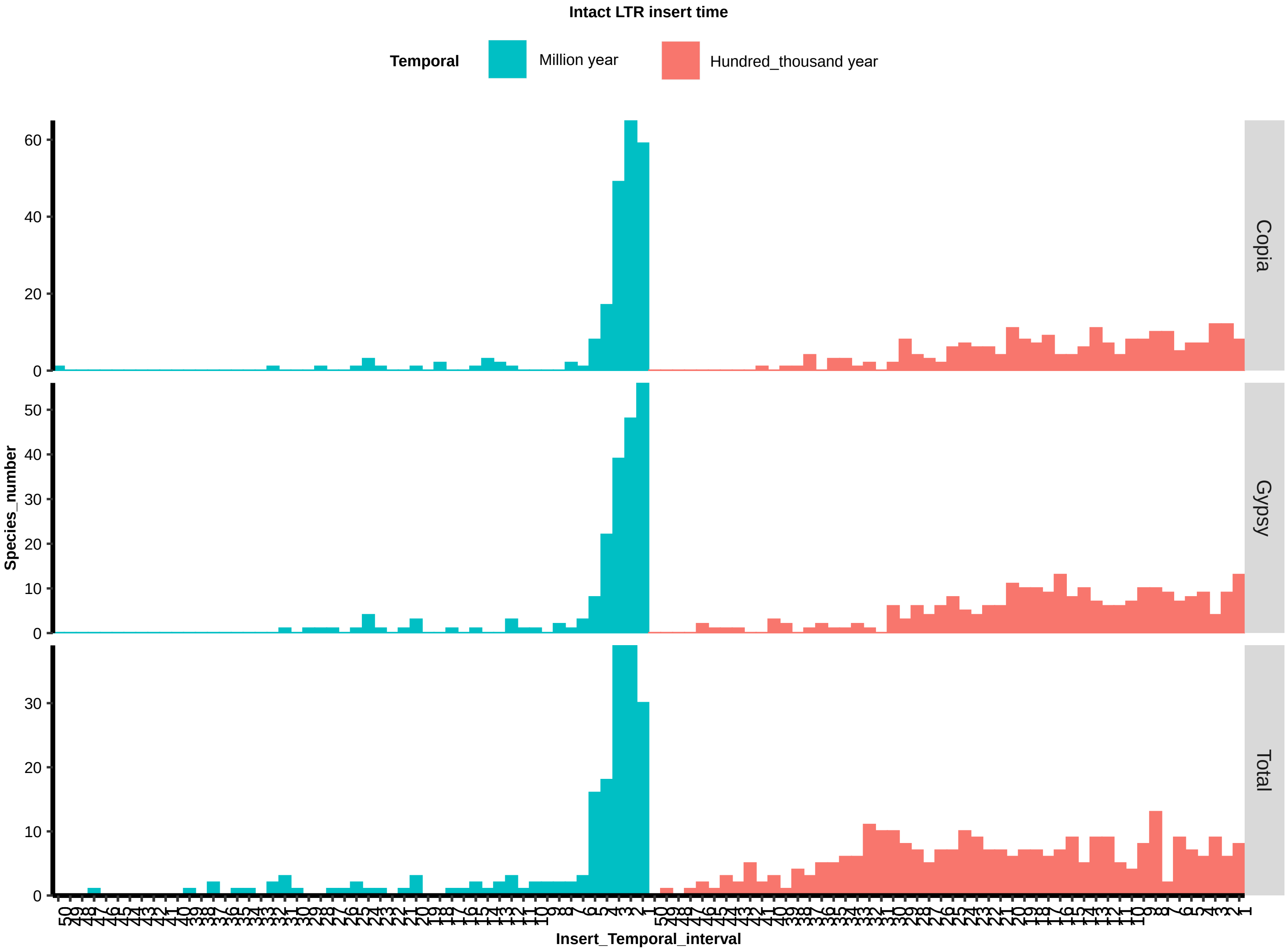

Supplementary Fig S18. The intact LTR insert time of every species.

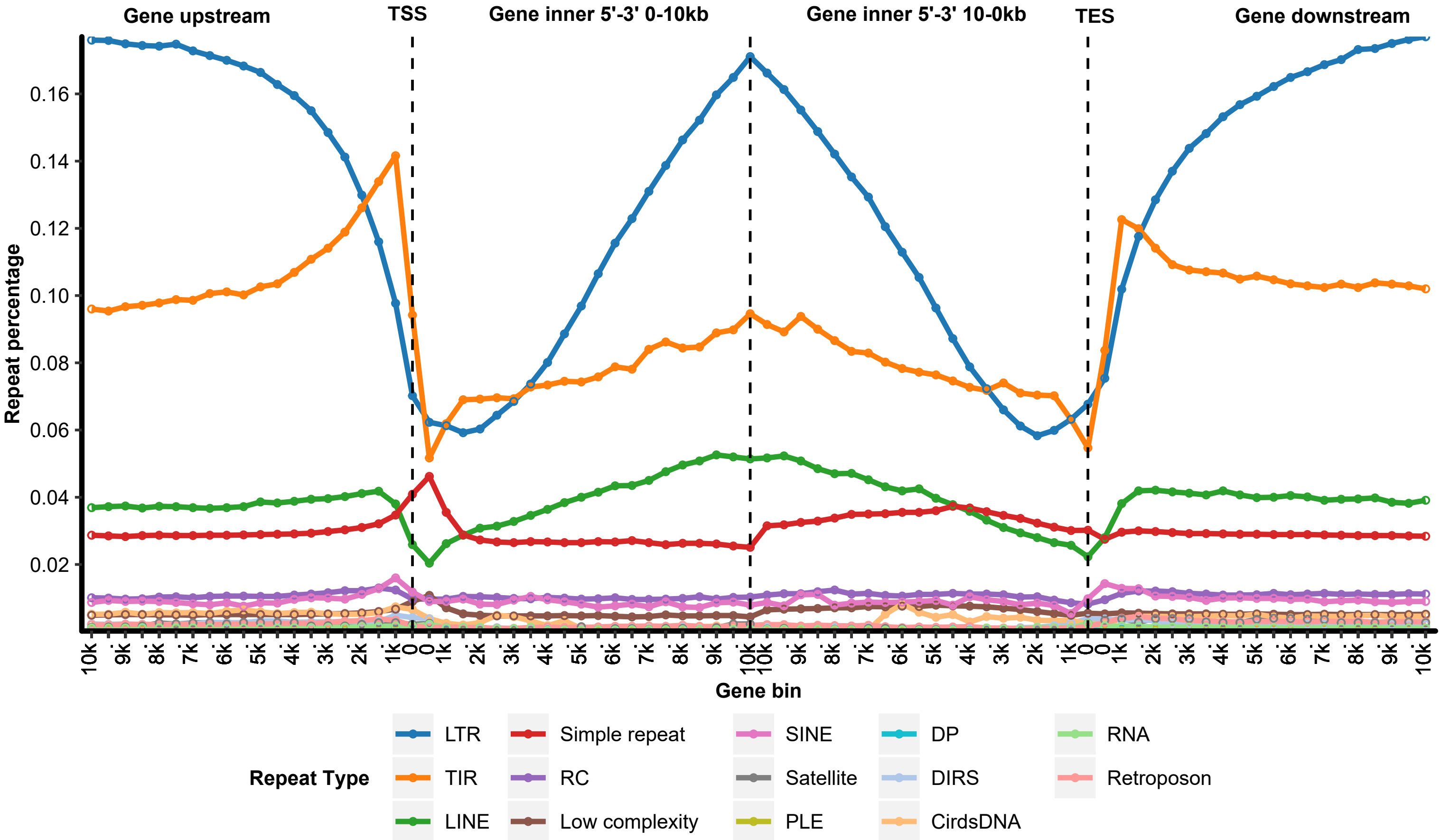

Supplementary Fig S19. Distributions of repeat sequences across gene bodies and flanking regions. The pecentage of repeats were calculated for each 0.5kb window. TSS, transcription start site; TES, transcription end site.

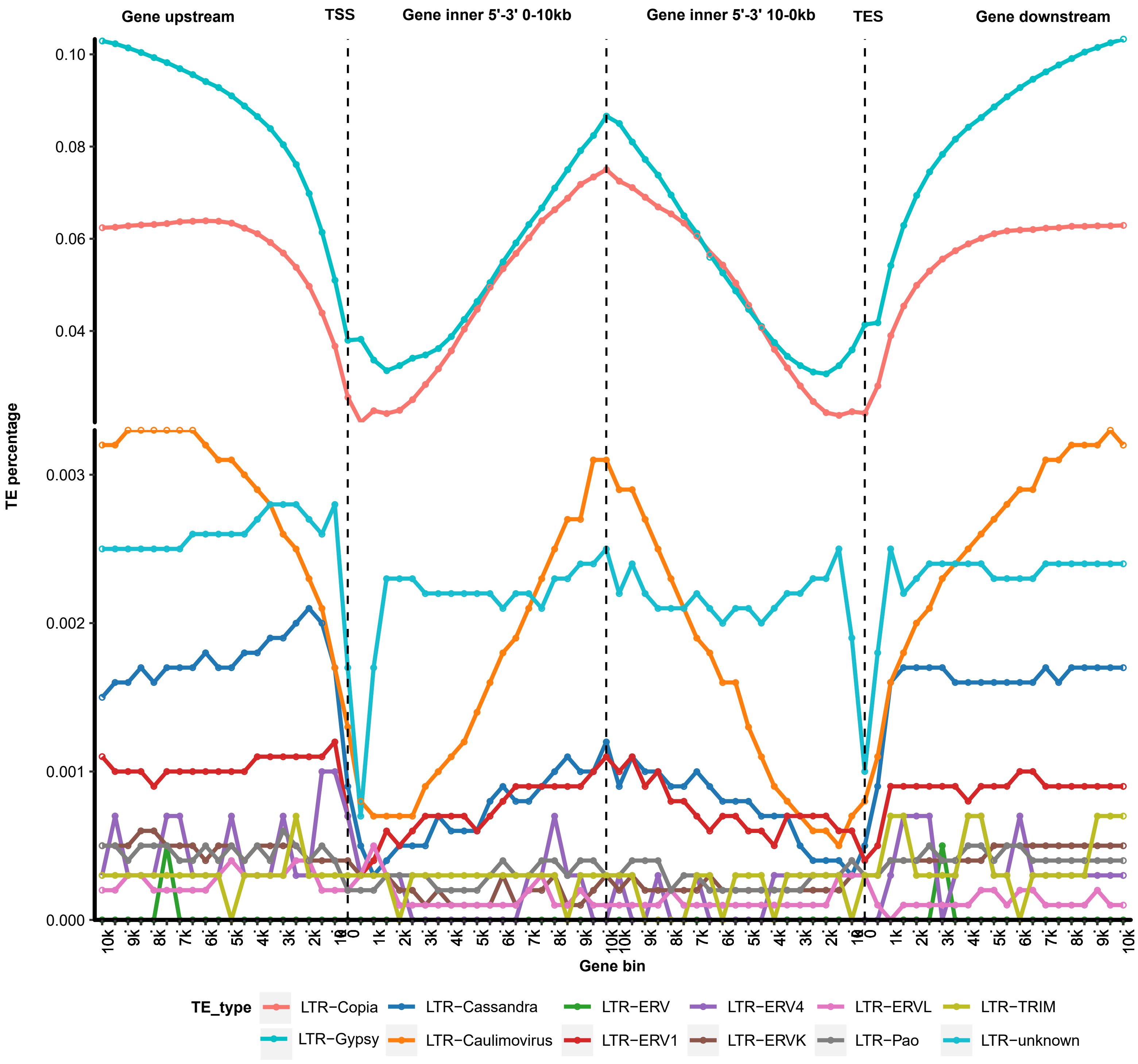

Supplementary Fig S20. Distributions of LTR across gene bodies and flanking regions. The pecentage of repeats were calculated for each 0.5kb window. TSS, transcription start site; TES, transcription end site.

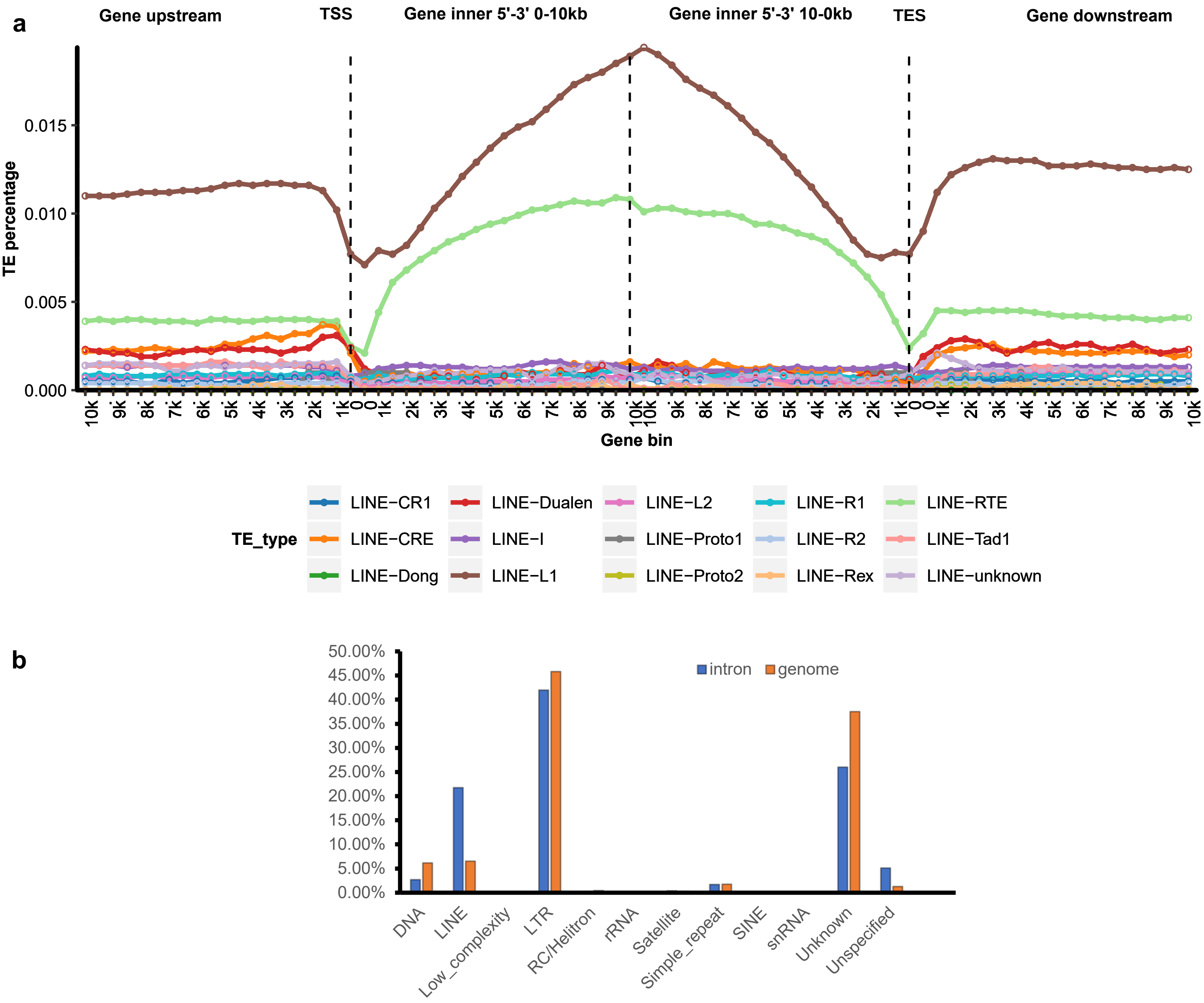

Supplementary Fig S21. (a) Distributions of LINE across gene bodies and flanking regions. The pecentage of repeats were calculated for each 0.5kb window. TSS, transcription start site; TES, transcription end site.
(b) *Adiantum capillus* repeat sequences in the genome and repeat sequences in intron.

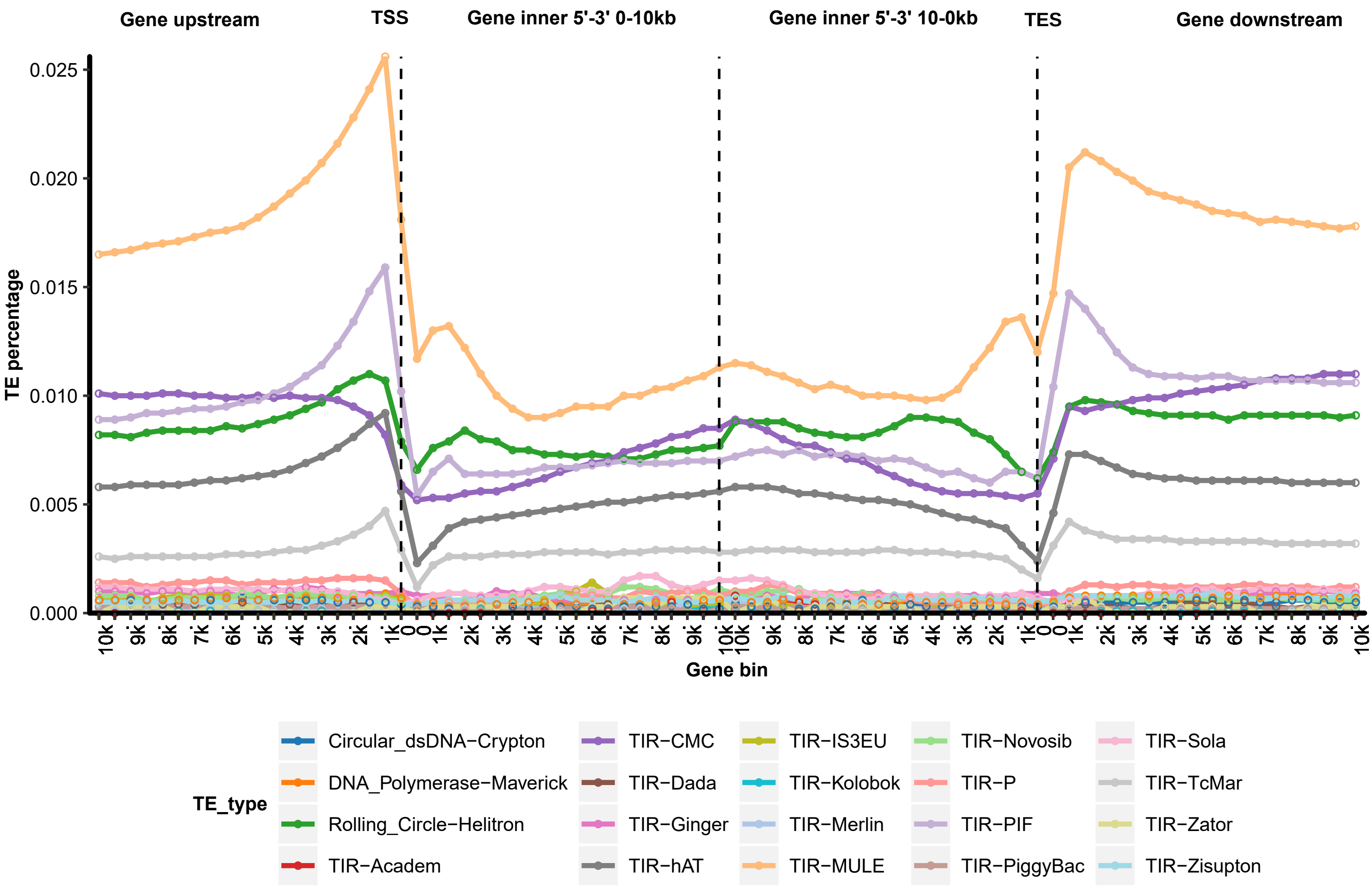

Supplementary Fig S22. Distributions of DNA transposon across gene bodies and flanking regions. The pecentage of repeats were calculated for each 0.5kb window. TSS, transcription start site; TES, transcription end site.
